# A non-invasive method to predict drought survival in Arabidopsis using quantum yield under light conditions

**DOI:** 10.1186/s13007-023-01107-w

**Published:** 2023-11-15

**Authors:** Thelma Y. Rico-Cambron, Elohim Bello-Bello, Octavio Martínez, Luis Herrera-Estrella

**Affiliations:** 1 National Laboratory of Genomics for Biodiversity (LANGEBIO), Unit of Advanced Genomics, CINVESTAV, Irapuato, Guanajuato, 36824 Mexico; 2 Plant Molecular and Cellular Biology Laboratory, Salk Institute for Biological Studies, La Jolla, CA 92037 USA; 3 Department of Plant and Soil Science, Institute of Genomics for Crop Abiotic Stress Tolerance, Texas Tech University, Lubbock, TX 79409 USA

**Keywords:** Chlorophyll *a* fluorometry, Drought, Fv’/Fm’, Handheld fluorometer, Non-invasive, Quantum yield

## Abstract

**Background:**

Survival rate (SR) is frequently used to compare drought tolerance among plant genotypes. While a variety of techniques for evaluating the stress status of plants under drought stress conditions have been developed, determining the critical point for the recovery irrigation to evaluate plant SR often relies directly on a qualitative inspection by the researcher or on the employment of complex and invasive techniques that invalidate the subsequent use of the tested individuals.

**Results:**

Here, we present a simple, instantaneous, and non-invasive method to estimate the survival probability of *Arabidopsis thaliana* plants after severe drought treatments. The quantum yield (QY), or efficiency of photosystem II, was monitored in darkness (Fv/Fm) and light (Fv’/Fm’) conditions in the last phase of the drought treatment before recovery irrigation. We found a high correlation between a plant’s Fv’/Fm’ value before recovery irrigation and its survival phenotype seven days after, allowing us to establish a threshold between alive and dead plants in a calibration stage. This correlation was maintained in the Arabidopsis accessions Col-0, Ler-0, C24, and Kondara under the same conditions. Fv’/Fm’ was then applied as a survival predictor to compare the drought tolerance of transgenic lines overexpressing the transcription factors ATAF1 and PLATZ1 with the Col-0 control.

**Conclusions:**

The results obtained in this work demonstrate that the chlorophyll *a* fluorescence parameter Fv’/Fm’ can be used as a survival predictor that gives a numerical estimate of the Arabidopsis drought SR before recovery irrigation. The procedure employed to get the Fv’/Fm’ measurements is fast, non-destructive, and requires inexpensive and easy-to-handle equipment. Fv’/Fm’ as a survival predictor can be used to offer an overview of the photosynthetic state of the tested plants and determine more accurately the best timing for rewatering to assess the SR, especially when the symptoms of severe dehydration between genotypes are not contrasting enough to identify a difference visually.

**Supplementary Information:**

The online version contains supplementary material available at 10.1186/s13007-023-01107-w.

## Background

Drought is considered one of the major threats to plant growth and crop productivity, imposing significant challenges to global agriculture and food security [1]. Immediate drought impacts on crop yield affect approximately 55 million people worldwide and cause annual economic losses reaching US$5.4 billion per year [2]. Therefore, there is an urgent need to develop new drought-resilient crop varieties and adopt farming practices that maximize crop yield under arid soil conditions. Several breeding programs and water management strategies have been implemented to achieve these sustainable agriculture goals, transform agri-food systems, and address pressing climate challenges [3]. To date, the genetic and physiological mechanisms underlying plant responses to low water availability have been widely studied [4, 5], providing plant breeders with information that facilitates the development of new plant varieties more tolerant to drought using both traditional methods, such as molecular plant breeding [6, 7], and cutting-edge technologies including genetic engineering [8, 9] and genome editing [10, 11]. Additionally, water-smart farming practices for mitigating water scarcity and improving water use efficiency are currently in the scope of farmers to ensure the long-term sustainability of agricultural systems. Such farming practices include the use of soil additives such as superabsorbent hydrogel [12, 13], nanoparticles [14, 15], biochar [16, 17], and plant growth-promoting rhizobacteria [18, 19], which enhance soil wettability and provide multiple benefits to improve crop development and soil health.

Despite implementing plant breeding programs and water management strategies to improve drought stress tolerance in crop plants, determining their effectiveness and suitability remains a major challenge. Therefore, it is necessary to establish more accurate techniques and methods to reliably measure plant drought tolerance and survival. In this regard, sophisticated equipment and platforms, such as portable photosynthesis systems and high-throughput multi-sensor gravimetric systems, have been designed to provide over-time profiles of plant status under different water regimes, combining the monitoring of parameters like stomatal conductance, transpiration rate, and water use efficiency [20–22], but the implementation of these platforms can result expensive and difficult to access. On the other hand, simpler drought-related indicators have been employed to assess the response of different plant genotypes to drought stress, including plant relative water content [23], biomass reduction [24], and ion leakage [25]. Nevertheless, employing these indicators is highly invasive, invalidating any posterior use of the tested plants. Among the main approaches for comparing drought tolerance between plant genotypes, plant viability is a straightforward way to test a plant’s capacity to survive severe drought stress conditions. This survival proof consists of withholding the water supply until most control individuals show signs of perishing. The survival rate (SR) after supplying recovery irrigation is considered a drought tolerance indicator [26–28]. However, this approach lacks effective methods to determine the optimal time for plant rewatering, relying on the experience of the researcher to observe qualitative drought-related plant characteristics, such as turgor loss, or requiring several replicates of the same experiment, resulting in a time-consuming, error-prone and resource-intensive process.

To cope with this concern, plant scientists have focused on photosynthesis, one of the first processes affected by drought, even at mild stress levels [29]. In this sense, the measurement of photosynthetic parameters related to chlorophyll *a* fluorescence has been employed to estimate the impact of drought on photosynthesis non-invasively. From these parameters, the most commonly used are 1) quantum yield (QY), which can be estimated under dark (maximum QY or Fv/Fm) and light conditions (operational QY or Fv’/Fm’); 2) rapid polyphasic chlorophyll *a* fluorescence transient (OJIP), and 3) non-photochemical quenching (NPQ) [30–34]. Specifically, QY represents the efficiency of photosystem II to employ the absorbed photon energy in photochemistry [35, 36]. The maximum QY, also called Fv/Fm, has been utilized as an indicator of plant health status under different abiotic stresses, such as heat [37], salinity [38], and drought [31, 39], to determine different levels of tolerance in crop plants. In Arabidopsis, Fv/Fm has been used as a quantitative parameter to predict plant survival and determine the time of death after a severe drought treatment [31]. A drawback of this technique is the requirement of dark adaptation of the plant samples, which can represent a hindrance for tracking experiments with a large number of samples or when the plant size is too small to use of the clamps or clips often required by chlorophyll fluorometers. Given this, exploring the behavior of dark-independent chlorophyll *a* parameters in plants exposed to drought stress could offer an alternative to overcome the limitations imposed by the need for a dark adaptation stage.

In this study, we compared the behavior of Fv/Fm and Fv’/Fm’ at a late stage of a drought stress treatment in *Arabidopsis thaliana* to evaluate the suitability of Fv’/Fm’ as a plant survival predictor, eliminating the necessity of an initial phase of dark adaptation. Our results demonstrate that Fv’/Fm’ measurements in light-adapted plants allow for predicting plant survival and viability after severe drought stress in a fast, precise, and non-invasive manner. Finally, this methodology was successfully applied to different Arabidopsis accessions and transgenic lines with varying degrees of drought tolerance, confirming its applicability and reproducibility in Arabidopsis research.

## Materials and methods

### Plant materials and growth conditions

In this work, four Arabidopsis accessions and two drought-tolerant transgenic lines were evaluated. The selected accessions were Columbia-0 (Col-0), used as the control line in all the experiments, Landsberg *erecta*-0 (Ler-0), C24 and Kondara. The transgenic lines were *pTCTP1::ATAF1-2* and *p35S::PLATZ1-8* (hereafter referred to as *ATAF1-2ox* and *PLATZ1-8ox*, respectively), which overexpress the genes *ARABIDOPSIS TRANSCRIPTION ACTIVATING FACTOR 1* (*ATAF1*, AT1G01720) and *PLANT A/T-RICH SEQUENCE- AND ZINC-BINDING PROTEIN 1* (*PLATZ1*, AT1G21000). These genes have been previously reported to improve the performance of Arabidopsis plants under low water availability [40, 41]. Arabidopsis accessions and the transgenic line *PLATZ1-8ox* were obtained from laboratory stock, while the line *ATAF1-2ox* was generated in this study as mentioned in the next section. Seeds were surface sterilized by adding 20% (v/v) commercial bleach and shaking for 10 min, followed by three rinses with sterile distilled water of 3 min each. After stratification for 48 h at 4 °C in darkness, seeds were sown in 100 × 15 mm Petri dishes (SYM Laboratorios, Puebla, Mexico) containing 25 ml of 0.1X Murashige and Skoog (MS) medium supplemented with 0.5% (w/v) sucrose, 3.5 mM MES, and 1% agar. Seedlings were grown for 14 days in growth chambers (Percival, IA, USA) at a constant temperature of 22 ± 2 °C and under a photoperiod of 16 h light/8 h dark with photosynthetically active radiation (PAR) of 80 ± 10 μmol m^− 2^ s^− 1^ supplied by white fluorescent lamps (Philips, Amsterdam, Netherlands). For seed propagation, 14-day-old seedlings were transferred to a soil mix composed of peat moss Sunshine® mix #3 (Sun Gro® Horticulture, Alberta, Canada), vermiculite (Vermi Radical, Guanajuato, Mexico) and perlite (Grupo Perlita, Durango, Mexico) in a relation 3:1:1. Soil mix was fertilized with 0.1X MS solution. Plants were allowed to complete their cycle in greenhouse.

### Generation of lines overexpressing *ATAF1*

An overexpression vector containing the *ATAF1* coding sequence under the control of the minimum constitutive promoter (0.3kb_pro_) from the *TRANSLATIONALLY CONTROLLED TUMOR PROTEIN 1* (*TCTP1*, AT3G16640) gene [42] was assembled using the Golden Gate Modular Cloning (MoClo) system [43, 44]. Briefly, *TCTP1* promoter and *ATAF1* coding sequence were amplified by PCR from genomic and complementary DNA, respectively, with the primers specified in Supplementary Table S1. The obtained sequences were cloned in L0 vectors to subsequently be joined to the *AtuOCS* terminator, included in the MoClo Plant Part (MPP) kit, in an L1 vector. This transcriptional unit was transferred to an L2 vector adding the bialaphos resistance cassette (MPP kit) as a selective marker (complete list of vectors in Supplementary Table S2). The final construction was introduced in *Agrobacterium tumefaciens* (strain GPV2260) to transform Col-0 plants by floral dip method [45]. Transgenic lines carrying *ATAF1* construction were selected in 0.1X MS medium containing 100 μM glufosinate-ammonium (Sigma-Aldrich, Darmstadt, Alemania); plants with an approximate 3:1 segregation rate, corresponding to single insertions, were chosen to produce homozygous lines.

### Analysis of *ATAF1* and *PLATZ1* expression by RT-qPCR

The expression level of the *ATAF1* and *PLATZ1* transgenes in the selected overexpressing lines was determined by RT-qPCR. Total RNA was isolated from 14-day-old seedlings using TRIzol reagent (Life Technologies, CA, USA). For RT-qPCR analysis, 500 ng of RNA were reverse-transcribed using the Super-Script III first-strand synthesis kit (Invitrogen, CA, USA) following the manufacturer’s instructions. Quantitative real-time PCR (qPCR) was performed on a Mic qPCR Cycler System (Bio Molecular Systems, Queensland, Australia) using the reagent SensiFast TM SYBR No-ROX Kit (Bioline, London, UK) and gene-specific primers (Supplementary Table S1). The qPCR settings were 95 °C for 2 min, followed by 40 cycles of 95 °C for 5 s, 65 °C for 10 s and 72 °C for 20 s. The relative expression level of *ATAF1* and *PLATZ1* genes was calculated using the housekeeping gene *ACTIN2* (*ACT2*) as a reference and the 2^−ΔCt^ method (Supplementary Fig. S1A, B).

### Drought stress assays

Drought stress assays consisted of withholding water supply to Arabidopsis plants until they reached a state of severe stress to evaluate their SR upon rehydration. The experiments were divided into two main stages: (1) a *calibration* stage to define the QY threshold between surviving and dead plants of Col-0 and the different Arabidopsis accessions; and (2) an *application* stage, where the identified QY threshold for Col-0 was applied to estimate its SR at different points and decide the specific point for adding recovery irrigation, getting an SR of this control line close to the rate estimated by previous recovery irrigation.

*Calibration stage*: For the calibration stage of Col-0 and the Arabidopsis accessions, square pots (8.40 × 6.35 × 8.89 cm) were filled with 40 g of dried soil (see *Plant materials and growth conditions* section). Pots were placed overnight in trays with 4L of 0.05X MS solution to allow the soil saturation by absorption. Solution excess was removed. Five seedlings (14 days old) were transferred per pot following the distribution shown in Supplementary Fig. S2C. Pots containing Arabidopsis plants were placed in a growth chamber (Percival, IA, USA) at 22 ± 2°C, with a photoperiod of 16 h light/8 h dark and a PAR intensity of 100 ± 10 μmol m^− 2^ s^− 1^. After ten days in well-watered conditions, pots for drought treatment were transferred to trays containing distilled water, where they were kept until reaching their saturation point. Then, water-saturated pots were placed on microfiber towels for 30 minutes to remove excess water. All pots were weighed and brought to the same weight (245 g) and finally, an initial water content was established (194 ml). Treated pots were left to dry, daily weighted, and adjusted with distilled water (approximately 1 to 5 ml per day) to get a uniform pot weight to keep a similar water content drop among them. Once soil water content dropped to 10%, water addition was withheld (from this point, pots maintained negligible differences in weight). When severe stress symptoms were observed (30 days of treatment), Fv’/Fm’ values started to be monitored. Recovery irrigation was applied at different times for experiments corresponding to the calibration stage to get a set of QY values between the average Fv’/Fm’ of well-watered plants and the minimum possible value for Fv’/Fm’ (0.00). The SR and thresholds were calculated seven days after recovery irrigation.

*Application stage*: To estimate the applicability of the method described here, we carried out a drought experiment with the *ATAF1-2ox* and *PLATZ1-8ox* transgenic lines at the Institute of Genomics for Crop Abiotic Stress Tolerance (IGCAST, Texas Tech University). Since some conditions changed for these experiments, such as soil, light source and controlled relative humidity, a small calibration experiment was performed with Col-0. For the drought stress assays for both calibration of Col-0 threshold and application stages, square pots (8.40 × 6.35 × 8.89 cm) were filled with 38 g of thoroughly dried growing substrate Sunshine® Mix #1 (Sun Gro® Horticulture, Alberta, Canada) and saturated with 100 mg/ml fertilizer All Purpose Plant Food (Miracle-Gro®). Five seedlings (14-days-old) were transferred to each pot and placed in a growth chamber (Conviron, CA, USA) at 22 ± 2°C, 16/8 h photoperiod, a PAR intensity of 100 ± 10 μmol m^− 2^ s^− 1^ supplied by LED lamps, and relative humidity of 45%. The drought treatment was conducted following the above-described protocol and until the plants showed severe stress symptoms (16 days of treatment). Recovery irrigation was applied according to the above-mentioned criteria for the calibration stage, but for the validation experiments, the probable SR of Col-0 was determined every hour by measuring the Fv’/Fm’ of each Col-0 plant and utilizing the corresponding threshold (Supplementary Fig. S1C). Recovery irrigation was added when a SR lower than 10% was estimated for Col-0 and, before rewatering, Fv’/Fm’ of *ATAF1-2ox* and *PLATZ1-8ox* was also measured. SR of the tested lines was evaluated seven days after recovery irrigation.

### Determination of maximum (Fv/Fm) and operational (Fv’/Fm’) QY in Arabidopsis plants

QY measurements were obtained using a handheld fluorometer PAR FluorPen FP 110/P (Photon Systems Instruments, Brno, Czech Republic) according to the settings recommended by the manufacturer: λ = 455 nm; flash-pulse: 900 μmol m^− 2^ s^− 1^, 30 μs; super-pulse: 2400 μmol m^− 2^ s^− 1^. Measurements were taken directly from the center of the rosette, pointing the fluorometer at 90°. For Fv/Fm, dark adaptation of the plants was carried out in a dark room (PAR ≤ 1 μmol m^− 2^ s^− 1^) for 15 min. To evaluate the effect of PAR on QY under well-watered conditions, PAR was adjusted to different intensities (20, 45, 70, 100, 140 and 185 μmol m^− 2^ s^−^1) by changing the number of fluorescent lamps in the growth chamber or placing the plants in a dark room (PAR ≤ 1 μmol m^− 2^ s^− 1^) in the case of Fv/Fm. PAR intensity was measured with the PAR sensor of the PAR FluorPen FP 110/P. Before QY measurements, plants were allowed to adapt to each PAR intensity for 15 min. For drought stress assays, once drought-treated plants showed stress symptoms, such as wilting, chlorosis, and dark-colored leaves, QY was monitored every hour.

### Estimation of survival rate

The parameter SR was calculated as *S/N x 100*, where *S* is the number of plants that survived the drought treatment seven days after recovery irrigation, and *N* is the total number of evaluated plants. To estimate SR before recovery irrigation, the Fv’/Fm’ values obtained during the monitoring of the control line (Col-0) were classified as PS (probable surviving) when they were equal to or higher than the threshold value, and as PD (probable dead) when they were lower than the threshold value. Estimated SR was calculated as *PS/N x 100*, where *PS* is the number of Fv’/Fm’ values classified as PS.

### Statistical analyses

All statistical analyses were done in R software (version 4.3.1, http://www.R-project.org). For QY and SRs comparisons, one-way-ANOVA, Student’s t-test, Wilcoxon signed-rank and Tukey’s HSD tests were conducted with a significance level of p-value < 0.01. Pearson and point-biserial correlations were performed to determine the interdependence of QY with PAR and SR, respectively, employing a p-value < 0.01. The thresholds were estimated by a Generalized Linear Model (GLM) for the surviving probability as a function of the observed Fv’/Fm’ parameter. We also performed a statistical validation for the method’s ability to predict plant survival as a function of Fv’/Fm’ (see Additional file 3). All graphs presented in this study were generated using the ggplot2 package (version 3.4.3).

## Results

### QY performance changes under different PAR intensities in well-watered conditions

**Fig. 1:**
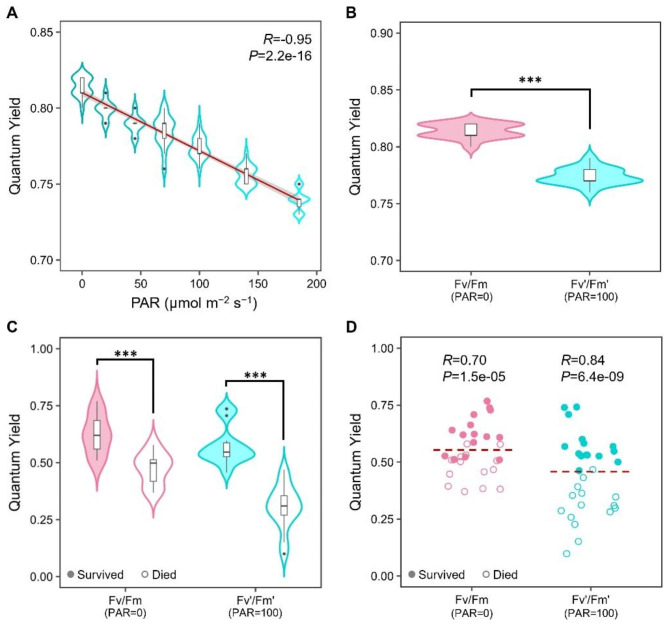
Analysis of QY in dark- and light-adapted Arabidopsis plants. **A**) QY measurements taken at different PAR intensities from Col-0 plants grown under well-watered conditions. Violin plots show the data distribution, median, 25^th,^ and 75th percentiles (n = 15 plants, three independent experiments). Redline, linear regression; gray area, 95% confidence region; *R*, Pearson correlation coefficient. **B**) Comparison of QY in dark- (Fv/Fm) and light-adapted (Fv’/Fm’, 100 μmol m^− 2^ s^− 1^) plants in well-watered conditions (n = 15 plants, three independent experiments). Asterisks, significant difference (Wilcoxon signed-rank test, *P* < 0.001. **C**) QY in dark- (Fv/Fm) and light-adapted (Fv’/Fm’, 100 μmol m^− 2^ s^− 1^) plants that survived or died after a severe drought treatment (n = 15 biological replicates, two independent experiments). Asterisks, significant differences (Student’s t-test, *P* < 0.001). **D**) Single QY measurements of drought-treated plants before recovery irrigation in the dark (Fv/Fm) and light conditions (Fv’/Fm’, 100 μmol m^− 2^ s^− 1^). Redline, threshold estimated by a GLM; *R*, point biserial correlation coefficient (n = 30 plants, two independent experiments)

To explore the impact of changes in PAR intensity on QY in Arabidopsis plants grown under well-watered conditions, we conducted measurements of the maximum (Fv/Fm) and the operational QY (Fv’/Fm’) in Col-0 plants adapted to different PAR intensities. Interestingly, we observed a progressive decline of QY values as PAR intensity increased, represented by the equation y = -0.0004x + 0.8101 and supported by a negative correlation coefficient between PAR and QY of -0.95 (Fig. 1A). The QY values of Col-0 plants oscillated from 0.81 to 0.74 at the lowest and highest tested PAR intensity (0 and 185 μmol m^− 2^ s^− 1^, respectively). A detailed analysis of the QY dynamics at the different tested PAR intensities indicated that, while the Fv/Fm of Col-0 plants was 0.81 at 0 μmol m^− 2^ s^− 1^ (darkness conditions), the Fv’/Fm’ at 20 and 185 μmol m^− 2^ s^− 1^ showed reductions of 1.2% (0.80) and 8.6% (0.74), respectively (Fig. 1A). At 100 μmol m^− 2^ s^− 1^, the PAR intensity generally used to grow Arabidopsis plants under standard conditions, Col-0 rosettes had an Fv’/Fm’ of 0.77, representing a significative reduction of 4.9% with respect to dark-adapted plants (Fig. 1A, B). These results indicate that interpolation curves can be created to compare QY values obtained at different PAR intensities and even calculate a sample’s maximum QY (Fv/Fm) from measurements of operational QY (Fv’/Fm’) without previous dark adaptation.

### Fv’/Fm’ as a potential indicator of plant survival rate in Arabidopsis

**Fig. 2:**
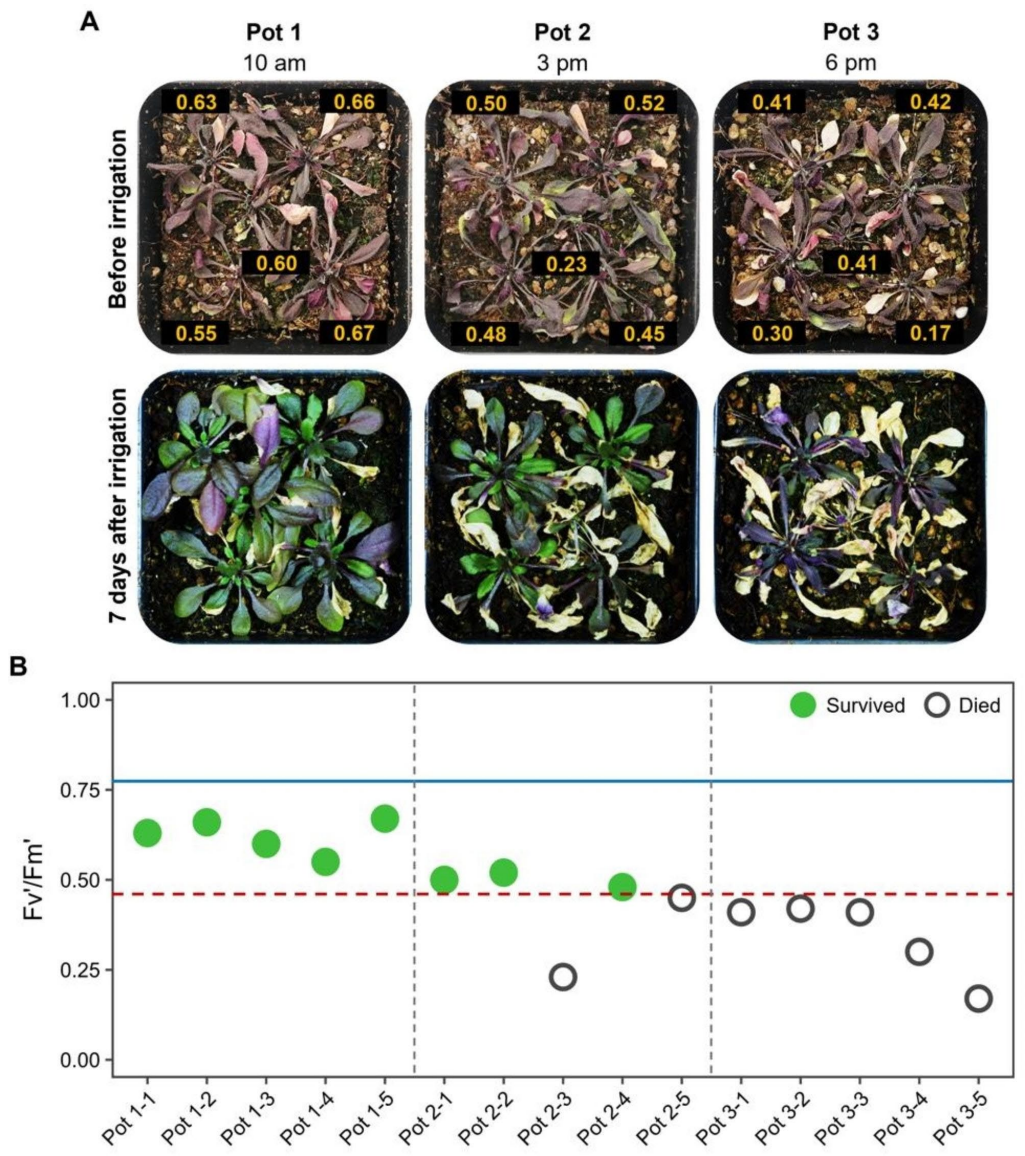
Relation between Fv’/Fm’ and drought survival in Arabidopsis Col-0 plants. (**A**) Drought-treated plants before (upper) and after (lower) receiving the recovery irrigation. The time at which each pot was irrigated is indicated. Labels, Fv’/Fm’ values. (**B**) QY in light conditions after a severe drought treatment. Dots represent the single Fv’/Fm’ measurements taken per plant before recovery irrigation. Blue line, Fv’/Fm’ in well-watered conditions; red line, threshold between surviving and dead plants established for Col-0

As mentioned in the introduction, Fv/Fm has been used as a survival predictor in Arabidopsis; however, dark adaptation becomes a problem when the assays are carried out with a large number of individuals or with small plants. To determine if QY in light-adapted samples (Fv’/Fm’) can also work as a survival predictor in Col-0 plants at the last phase of a terminal drought treatment (before recovery irrigation), we decided to carry out an initial experiment in 30 Arabidopsis plants to get insights about QY performance under drought conditions. For this experiment, Fv/Fm and Fv’/Fm’ values for all plants were recorded before applying the recovery irrigation, and, seven days after, these values were compared between surviving and dead plants. As shown in Fig. 1C, both types of QY (Fv/Fm and Fv’/Fm’) significantly differed between surviving and dead plants. While in dark adaptation, QY dropped 22.6% between surviving and dead individuals (Fv/Fm = 0.62 and 0.48, respectively), a higher difference of 46.6% was observed in light adaptation (Fv’/Fm’=0.58 and 0.31, respectively). A detailed analysis of QY data showed that Fv’/Fm’ has a stronger correlation (*R* = 0.84) with the plant survival phenotypes (surviving/dead) than Fv/Fm (*R* = 0.70). Further analysis using a GLM of the entire dataset of QY values (n = 30) showed that a putative Fv’/Fm’ threshold of 0.46 allowed predicting SR in Col-0 with an efficiency of 97% in comparison with the threshold for Fv/Fm (0.55), that only has a predictive power of 80% (Fig. 1D). These results suggest that Fv’/Fm’ can be used as a rapid, non-invasive survival indicator after severe drought treatments. As can be observed in Fig. 2, although there are no evident differences in drought stress symptoms among treated plants (wilting, dark-colored leaves, chlorosis, etc.), the established threshold for Col-0 can accurately separate surviving plants from dead ones. Moreover, as observed in the last day of treatment, the FV’/FM’ and plant survival drop rapidly (Fig. 2), highlighting the importance of monitoring QY throughout the day.

### Fv’/Fm’ works as a drought survival predictor among different Arabidopsis accessions

**Fig. 3:**
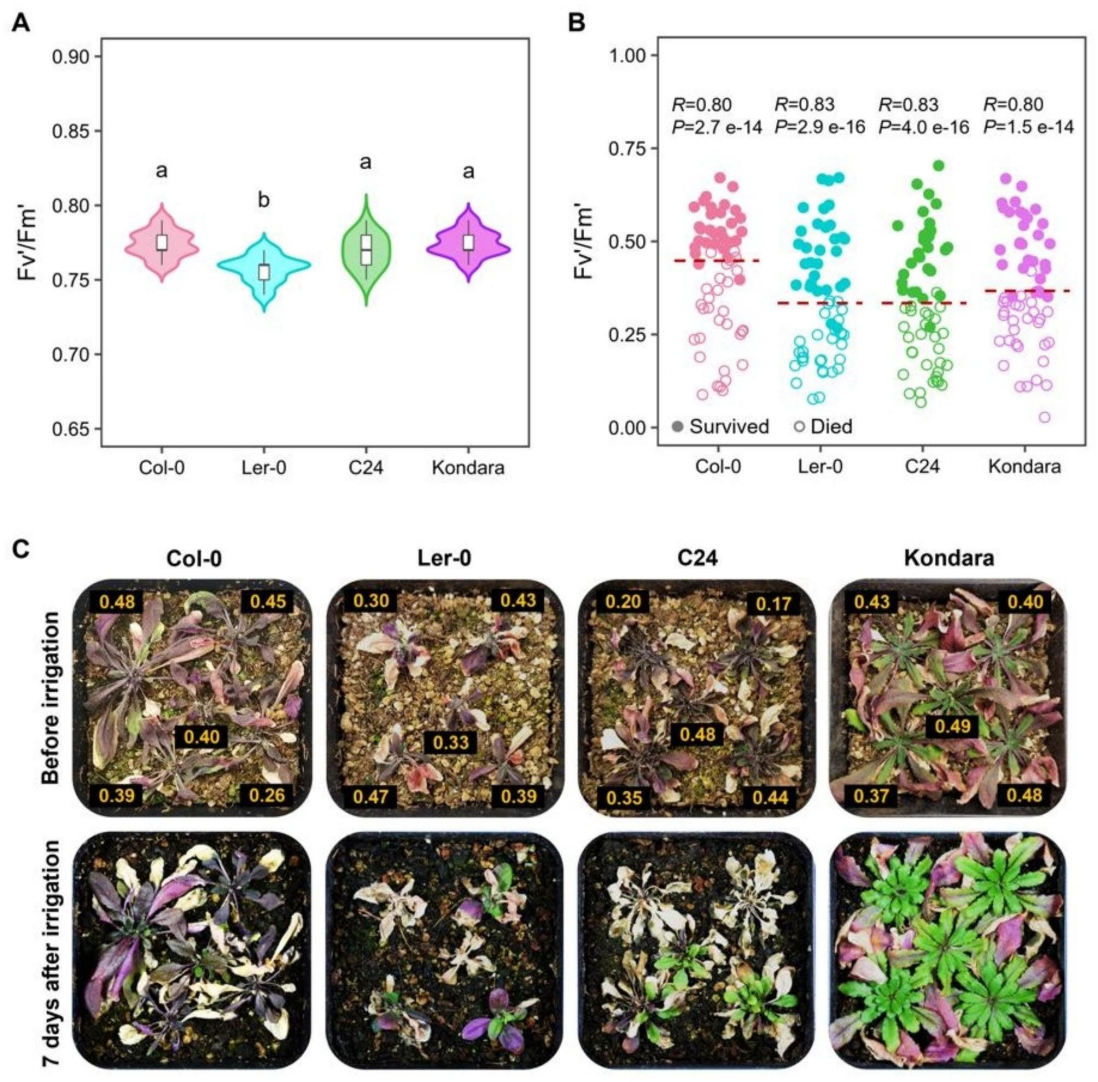
Fv’/Fm’ analysis among different Arabidopsis accessions under drought conditions. (**A**) Fv’/Fm’ values from Col-0, Ler-0, C24, and Kondara accessions in well-watered conditions. Letters, significant differences (n = 18 plants, three independent experiments, one-way ANOVA and Tukey’s HSD test, *P* < 0.01). (**B**) Single measurements of drought-treated plants before recovery irrigation. Redline, threshold estimated by a GLM with a training sample size of 50 measurements and validated in the remaining 10 measurements (1000 iterations); *R*, point biserial correlation coefficient (n = 60 plants, three independent experiments). (**C**) Adult plants from different Arabidopsis accessions submitted to a severe drought treatment before (upper) and after (lower) receiving a recovery irrigation. Labels, Fv’/Fm’ values

To examine if Fv’/Fm’ works as a drought survival predictor among different Arabidopsis genetic backgrounds, we evaluated Fv’/Fm’ performance in the Arabidopsis accessions Col-0, Ler-0, C24, and Kondara under both well-watered and severe drought conditions. Under a well-watered regime, C24 and Kondara accessions didn’t show significant differences in Fv’/Fm’ with respect to Col-0 (0.77); however, we found a small but significant reduction of 1.3% (0.76) in the Arabidopsis accession Ler-0 (Fig. 3A). For evaluation under drought stress conditions, we exposed the selected accessions to a period of water deprivation until the plants showed severe symptoms of stress. All the tested accessions presented a high correlation between Fv’/Fm’ and survival phenotype (*R* > 0.80). The thresholds between surviving and dead plants for each accession were determined and validated by a GLM analysis that calculates the survival probability in function of Fv’/Fm’, where 50 from a total of 60 measurements per accession were used as *‘training’* to estimate the predictive power of Fv’/Fm’ in the 10 remaining measurements (1000 iterations) (Additional file 3). This model set the threshold for Col-0 as 0.45 with a predictive power of 90%, i.e., just 0.01 below the first calculated threshold, corroborating that Fv’/Fm’ effectively can be used as a survival predictor in Col-0 and that a minimum of 30 samples is enough to set a threshold that efficiently separated viable plants from dead ones. The thresholds for Ler-0, C24, and Kondara were established as 0.33, 0.33 and 0.37, respectively, and, by performing a detailed statistical validation of the method, we confirmed that the prediction of plant survival in all the tested Arabidopsis accessions gives an approximate 90% of correct predictions (Fig. 3B, C, Additional file 3). These results indicate that Fv’/Fm’ has a wide application as a survival predictor among different Arabidopsis accessions.

### Application of Fv’/Fm’ as a survival predictor in the evaluation of Arabidopsis transgenic lines grown under drought stress

**Fig. 4:**
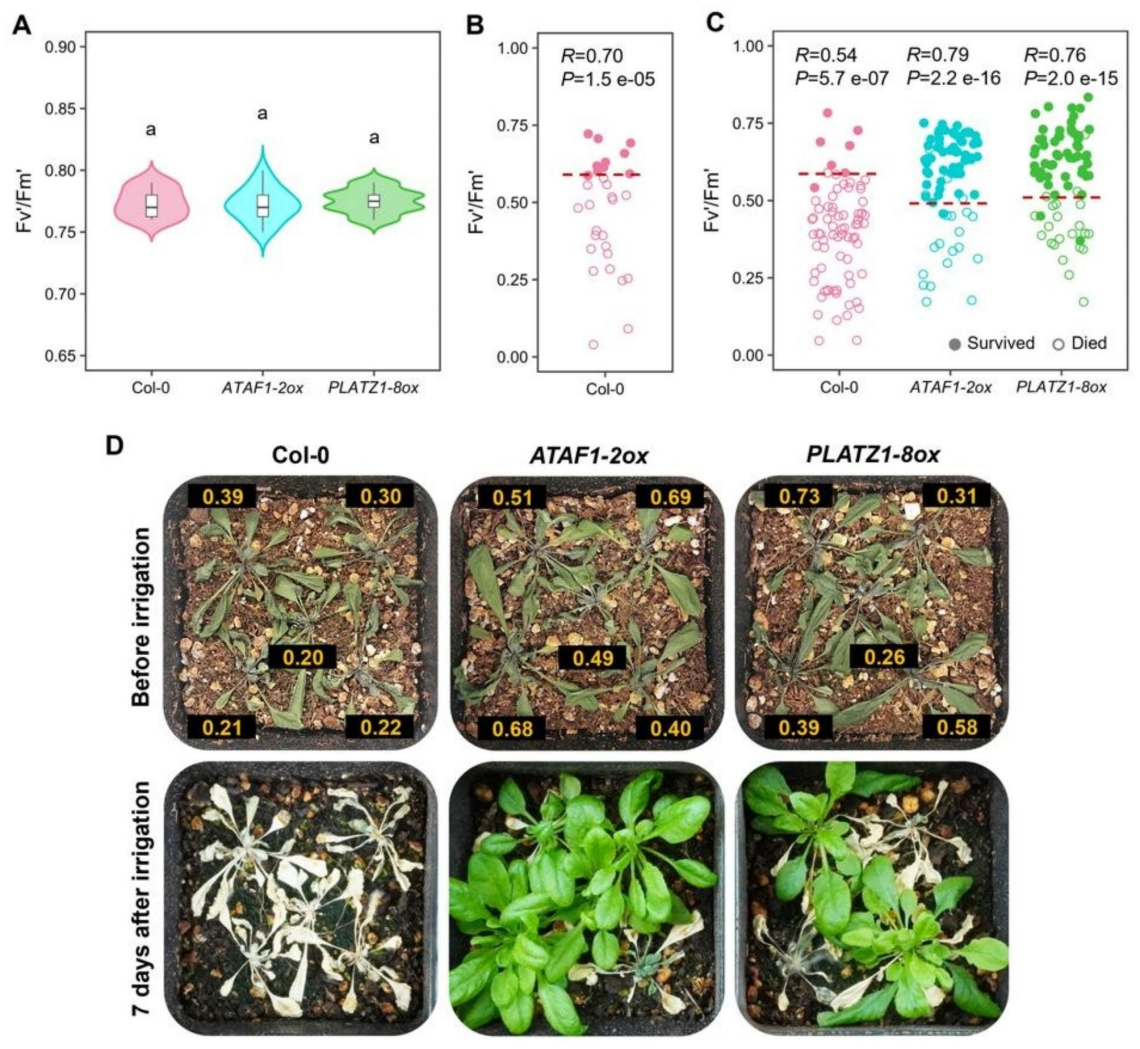
Correlation between Fv’/Fm’ and drought survival in drought-tolerant Arabidopsis transgenic lines. (**A**) Fv’/Fm’ exhibited by Col-0, *ATAF1-2ox*, and *PLATZ1-8ox* plants in well-watered conditions. Letters, significant differences (n = 18 plants, three independent experiments, one-way ANOVA and Tukey’s HSD test, *P* < 0.01). (**B**) Calibration of the threshold for Col-0 with single Fv’/Fm’ measurements of drought-treated Col-0 individuals before recovery irrigation. Redline, threshold estimated by a GLM; *R*, point biserial correlation coefficient (n = 30 plants, three independent experiments). (**C**) Single measurements of drought-treated plants before receiving a recovery irrigation. Redline, threshold estimated by a GLM; *R*, point biserial correlation coefficient (n = 75 plants, three independent experiments). Legend in C) also applies to B). (**D**) Col-0, *ATAF1-2ox* and *PLATZ1-8ox* plants under a severe drought treatment before (upper) and after (lower) receiving a recovery irrigation. Labels, Fv’/Fm’ values

To further explore the applicability of Fv’/Fm’ as a drought survival predictor that estimates the SR of a specific line before recovery irrigation, we decided to assay the ability of two transgenic lines (*ATAF1-2ox* and *PLATZ1-8ox*) to survive after a severe drought treatment comparing their SR at a critical point (SR ≤ 10%) for Col-0 plants (control line). These experiments were carried out in the IGCAST of Texas Tech University; therefore, it was necessary to determine if the behavior of Col-0 under well-watered and drought conditions in the new laboratory would be maintained as previously observed. Interestingly, under well-watered conditions, Col-0 kept the same Fv’/Fm’ value as the above experiments, 0.77, and there was no difference with *ATAF1-2ox* and *PLATZ1-8ox* (Fig. 4A). However, when Col-0 was submitted to drought treatment in a calibration stage to calculate the threshold for estimating SR, the drying period until the perishing state decreased from 30 to 16 days and, in the same way, the threshold between surviving and dead plants determined in the previous experiments increased from 0.48 to 0.59 (Fig. 4B), indicating that drying speed affects the QY state at which Col-0 can remain viable.

In the application stage, where Col-0 was compared with the lines *ATAF1-2ox* and *PLATZ1-8ox*, the new threshold was used to get an estimated value of the possible SR of Col-0 every hour during the last day of the drought treatment. Recovery irrigation was supplied once the estimated Col-0 SR fell below the survival limit of 10% (Supplementary Fig. S1C). Although the correlation between Fv’/Fm’ and Col-0 survival in these experiments was lower (*R* = 0.70) than the previously obtained experiment (*R* = 0.84), the use of this new threshold for Col-0 (0.59) (Fig. 4B) to determine the best time for recovery irrigation gave as result a Col-0 SR of 9.3% (Supplementary Fig. S1D), that is, lower than 10% as expected, with a predictive accuracy of 97% (Fig. 4C, D). At the same time, we observed significantly higher SRs for the *ATAF1-2ox* and *PLATZ1-8ox* transgenic lines (73.3 and 69.3%, respectively) (Supplementary Fig. S1D). In addition, since the Fv’/Fm’ values taken from *ATAF1-2ox* and *PLATZ1-8ox* also had a high correlation index with the survival of these lines (*R* = 0.79 and 0.76), it was possible to calibrate efficient thresholds by a GLM for both transgenic lines, being these 0.49 and 0.50 with predictive efficiencies of 92 and 93%, respectively (Fig. 4C, D). This suggests that Fv’/Fm’ can be used to estimate, before recovery irrigation, the SR of different Arabidopsis lines after a severe drought treatment.

## Discussion

Plant phenotyping for drought tolerance is crucial for developing drought-resilient crop varieties and establishing farming practices to help alleviate the havoc caused by climate change. This requires the generation of robust, non-destructive phenotyping methodologies to quantitatively evaluate the tolerance level among different plant genotypes. SR has been widely used as one of the most direct parameters to assay the levels of stress plant tolerance under severe drought conditions. Nevertheless, the main inconvenience related to its use is the need for reliable methods to determine the optimal time to add the recovery irrigations and avoid the disruption of different parts of the tested plants or even whole individuals.

Our results showed that Fv/Fm and Fv’/Fm’ averages of dying plants are considerably lower than those of surviving plants after severe drought recovery. Moreover, we found that the individual measurements of Fv’/Fm’ take a broader spread that permits better separating the values of surviving and dead plants, allowing us to more accurately determine a threshold that predicts the SR of drought-treated plants. In previous works, photosynthetic parameters like chlorophyll *a* fluorescence and Fv/Fm, estimated by image analysis, were reported as survival predictors. In these experiments, measurements were recorded before dawn since these parameters require a stage of dark adaptation to be determined [31, 32]. Nevertheless, drought survival can change drastically during the light part of the photoperiod (Fig. 2, Supplementary Fig. S1C), making the continuous monitoring of QY during the day imperative. Dark adaptation under light conditions can be achieved by placing the plants in a light isolation chamber [46], using covers like aluminum foil sheets [32], or with specialized leaf clamps or clips [47, 48], which increase the risk of damage to the tissues and make this a time-consuming task. In a critical point of drought treatment, when measurements are needed every hour, time for dark adaptation is unsuitable when many samples need to be processed. Measurement of Fv’/Fm’ with the PAR FluorPen FP110/P takes only 3 s and offers the advantage of providing a survival prediction without dark adaptation, allowing the handling of many samples per experiment. However, one point to consider when using photosynthetic parameters in light-adapted plants is the changes these could present depending on PAR intensity. This leaves the need to make all the measurements at a fixed PAR, which can constitute a challenge for evaluations under field or greenhouse conditions. To overcome this, some research groups apply actinic light at a defined intensity with the fluorometer device to simulate light adaptation [28], abolishing the advantage discussed here on time saving. Our results showed that the QY curve in Arabidopsis is strongly and negatively correlated to PAR intensity (Fig. 1A). Similar results have been reported in tomato (*Solanum lycopersicum* Mill.) and rice (*Oryza sativa* L.) under controlled conditions [49, 50] and for other photosynthetic parameters, such as F690/F730 [51]. This opens the possibility of generating curves of the Fv’/Fm’ trend with respect to changes in PAR, allowing researchers to interpolate and compare the Fv’/Fm’ values obtained at different PAR intensities. Nonetheless, several measurements could be required for more accurate estimations since data variation around the regression line could reduce the predictive precision (Fig. 1A).

Plant survival is assessed as the ability of a plant to resume its physiological functions and produce new tissues and organs after a dreadful stress event; hence, survival closely depends on the ability of the plant to ensure the viability of the meristems [52]. Meristematic cells cannot photosynthesize since they lack functional chloroplasts, but the immediate cells, such as leaf primordium, maintain a high photosynthetic activity to provide photosynthates to meristems [53]. Furthermore, under stress conditions, plants activate senescence programs in old leaves that prioritize the survival of the youngest ones [54], which are proximal to the meristematic zone. For this reason, in plants with rosette morphology, such as Arabidopsis, which maintain their survival priority in the center of the plant, it is preferable to choose the zones closest to meristems when using probe devices like PAR FluorPen FP 110/P that can only take measurements in reduced areas. The probe of PAR FluorPen FP 110/P is wide enough to cover all the shoot apical meristem and the surrounding area when pointed to the center of the rosette perpendicularly, making it possible to obtain measurements that can be accurately correlated with the probability of a plant to survive.

Different Arabidopsis accessions and genetically modified lines have been used to unveil the biological mechanisms behind drought tolerance. One of the most widely used parameters to measure drought tolerance among different lines is SR; however, its main limitation is the ambiguity of the criteria to determine the time for supplying the recovery irrigation, which can lead to inaccurate results with high variability. We demonstrated that Fv’/Fm’ is a good survival predictor in different Arabidopsis accessions and transgenic lines, suggesting its general application to estimate the SR of the line used as a reference by monitoring its Fv’/Fm’ at critical points of drought treatment. The researcher will determine the criteria for resuming irrigation depending on what is pretended to be observed, and this can be repeated through independent experiments to get reproducible results. In this regard, our results showed that using Fv’/Fm’ to estimate the SR of the control line (Col-0), it was possible to resume irrigation at an adequate time to replicate the results previously reported for *PLATZ1* and *ATAF1*, i.e., constitutive expression of these genes increases the ability of Arabidopsis to survive under drought conditions in which Col-0 presents low SR [40, 41].

The results of this study showed that the tested accessions Ler-0, C24 and Kondara, and the transgenic lines *ATAF1-2ox* and *PLATZ1-8ox* presented considerably lower thresholds in comparison with Col-0, indicating that, under severe levels of drought stress, they can maintain viability at lower photosynthetic rates than Col-0. This could conserve a tight relation with the high drought tolerance previously reported for C24 [55] and the lines overexpressing the genes *ATAF1* and *PLATZ1* [40, 41]. In addition, our results suggest that the rate of soil water loss also influences the Fv’/Fm’ value at which a plant can remain viable. When the drying time of the drought treatment decreased from 30 to 16 days, Col-0 plants could not survive at Fv’/Fm’ values under 0.59, which could be putatively attributed to the reduction of the window of time to generate adaptation responses. Altogether, these results indicate that the ability of a plant to remain viable until low QY values is closely linked to its capacity to activate efficient responses to cope with the stress and protect the photosynthetic machinery. This strongly depends on the genotype and the time to respond to the stress. Further experiments are being carried out to validate this hypothesis.

## Conclusion

In this study, we report a simple method based on measuring the chlorophyll *a* fluorescence parameter Fv’/Fm’ to predict SR among Arabidopsis plant genotypes subjected to severe drought stress after a calibration stage where the threshold between alive and dead samples is set up. This parameter provides a quantitative criterion that highly correlates with loss of plant viability and can be quickly measured with a PAR FluorPen FP 110/P, eliminating the time and effort for dark adaptation of samples. The PAR FluorPen FP 110/P is an inexpensive and easy-to-handle probe-based fluorometer that allows getting large amounts of data in a short time. Moreover, the portability of this device and the conditions required to measure Fv’/Fm’ allow its application under greenhouse and field conditions. Overall, the results presented here demonstrate the suitability of Fv’/Fm’ as a rapid, non-invasive drought survival predictor that can complement existing methodologies to accurately determine the drought tolerance level in different Arabidopsis genotypes.

### Electronic supplementary material

Below is the link to the electronic supplementary material.

**Additional file 1: Supplementary Table S1**: Primers used in this study. **Supplementary Table S2**: Golden Gate MoClo Vectors used in this study

**Additional file 2: Supplementary Figure S1**: Relative expression and drought survival analysis of two Arabidopsis transgenic lines. **Supplementary Figure S2**: Plants of different Arabidopsis genotypes grown under well-watered conditions

**Additional file 3**: Detailed statistical validation of the method

## Data Availability

The datasets analyzed during the current study are available from the corresponding author upon reasonable request.

## Abbreviations

GLM: Generalized Linear Model
PAR: Photosynthetic Active Radiation
QY: Quantum Yield
SR: Survival rate

## References

[1] Wang Z, Li J, Lai C, Wang RY, Chen X, Lian Y (2018). Drying tendency dominating the global grain production area. Glob Food Sec.

[2] Ligtvoet W, Bouwman A, Knoop J, de Bruin S, Nabielek K, Huitzing H (2018). The geography of future water challenges.

[3] Ahluwalia O, Singh PC, Bhatia R (2021). A review on drought stress in plants: implications, mitigation and the role of plant growth promoting rhizobacteria. Resour Environ Sustain.

[4] Yang X, Lu M, Wang Y, Wang Y, Liu Z, Chen S (2021). Response mechanism of plants to drought stress. Horticulturae.

[5] Gupta A, Rico-Medina A, Caño-Delgado AI (2020). The physiology of plant responses to drought. Science.

[6] Wani SH, Choudhary JR, Choudhary M, Rana M, Gosal SS (2020). Recent advances in genomics assisted breeding for drought stress tolerance in major cereals. J Cereal Res.

[7] Dixit S, Yadaw RB, Mishra KK, Kumar A (2017). Marker-assisted breeding to develop the drought-tolerant version of Sabitri, a popular variety from Nepal. Euphytica.

[8] Khan S, Anwar S, Yu S, Sun M, Yang Z, Gao ZQ (2019). Development of drought-tolerant transgenic wheat: achievements and limitations. Int J Mol Sci.

[9] Gao SQ, Chen M, Xu ZS, Zhao CP, Li L, Xu HJ (2011). The soybean GmbZIP1 transcription factor enhances multiple abiotic stress tolerances in transgenic plants. Plant Mol Biol.

[10] Joshi RK, Bharat SS, Mishra R (2020). Engineering drought tolerance in plants through CRISPR/Cas genome editing. 3 Biotech.

[11] Roca Paixão JF, Gillet FX, Ribeiro TP, Bournaud C, Lourenço-Tessutti IT, Noriega DD (2019). Improved drought stress tolerance in Arabidopsis by CRISPR/dCas9 fusion with a histone AcetylTransferase. Sci Rep.

[12] Saha A, Sekharan S, Manna U (2020). Superabsorbent hydrogel (SAH) as a soil amendment for drought management: a review. Soil Tillage Res.

[13] Tomášková I, Svatoš M, Macků J, Vanická H, Resnerová K, Čepl J (2020). Effect of different soil treatments with hydrogel on the performance of drought-sensitive and tolerant tree species in a semi-arid region. Forests.

[14] Saxena R, Tomar RS, Kumar M (2016). Exploring nanobiotechnology to Mitigate Abiotic stress in crop plants. J Pharm Sci Res.

[15] Sun L, Song F, Zhu X, Liu S, Liu F, Wang Y (2021). Nano-ZnO alleviates drought stress via modulating the plant water use and carbohydrate metabolism in maize. Arch Agron Soil Sci.

[16] Mansoor S, Kour N, Manhas S, Zahid S, Wani OA, Sharma V (2021). Biochar as a tool for effective management of drought and heavy metal toxicity. Chemosphere.

[17] Zhang Y, Ding J, Wang H, Su L, Zhao C (2020). Biochar addition alleviate the negative effects of drought and salinity stress on soybean productivity and water use efficiency. BMC Plant Biol.

[18] Camaille M, Fabre N, Clément C, Barka EA (2021). Advances in wheat physiology in response to drought and the role of plant growth promoting rhizobacteria to trigger drought tolerance. Microorganisms.

[19] Lim JH, Kim SD (2013). Induction of drought stress resistance by multi-functional PGPR *Bacillus licheniformis* K11 in pepper. Plant Pathol J.

[20] Zait Y, Ferrero-Serrano Á, Assmann SM (2021). The α subunit of the heterotrimeric G protein regulates mesophyll CO2 conductance and drought tolerance in rice. New Phytol.

[21] Dalal A, Shenhar I, Bourstein R, Mayo A, Grunwald Y, Averbuch N (2020). A telemetric, gravimetric platform for real-time physiological phenotyping of plant–environment interactions. J Vis Exp.

[22] Halperin O, Gebremedhin A, Wallach R, Moshelion M (2017). High-throughput physiological phenotyping and screening system for the characterization of plant–environment interactions. Plant J.

[23] Sapes G, Sala A (2021). Relative water content consistently predicts drought mortality risk in seedling populations with different morphology, physiology and times to death. Plant Cell Environ.

[24] Pimratch S, Jogloy S, Vorasoot N, Toomsan B, Patanothai A, Holbrook CC (2008). Relationship between biomass production and nitrogen fixation under drought-stress conditions in peanut genotypes with different levels of drought resistance. J Agron Crop Sci.

[25] Roy R, Agrawal V, Gupta SC (2009). Comparison of drought-induced polypeptides and ion leakage in three tomato cultivars. Biol Plant.

[26] Nishiyama R, Watanabe Y, Fujita Y, Le DT, Kojima M, Werner T (2011). Analysis of cytokinin mutants and regulation of cytokinin metabolic genes reveals important regulatory roles of cytokinins in drought, salt and abscisic acid responses, and abscisic acid biosynthesis. Plant Cell.

[27] Catala R, Ouyang J, Abreu IA, Hu Y, Seo H, Zhang X (2007). The *Arabidopsis* E3 SUMO ligase SIZ1 regulates plant growth and drought responses. Plant Cell.

[28] Qin F, Kakimoto M, Sakuma Y, Maruyama K, Osakabe Y, Tran LSP (2007). Regulation and functional analysis of *ZmDREB2A* in response to drought and heat stresses in *Zea mays* L. Plant J.

[29] Siddique Z, Jan S, Imadi SR, Gul A, Ahmad P, Ahmad P (2016). Drought stress and photosynthesis in plants. Water stress and crop plants: a sustainable Approach.

[30] Sommer SG, Han E, Li X, Rosenqvist E, Liu F (2023). The chlorophyll fluorescence parameter Fv/Fm correlates with loss of grain yield after severe drought in three wheat genotypes grown at two CO2 concentrations. Plants.

[31] Woo NS, Badger MR, Pogson BJ (2008). A rapid, non-invasive procedure for quantitative assessment of drought survival using chlorophyll fluorescence. Plant Methods.

[32] Guadagno CR, Ewers BE, Speckman HN, Aston TL, Huhn BJ, Devore SB (2017). Dead or alive? Using membrane failure and chlorophyll *a* fluorescence to predict plant mortality from drought. Plant Physiol.

[33] Dunić JA, Mlinarić S, Pavlović I, Lepeduš H, Salopek-Sondi B (2023). Comparative analysis of primary photosynthetic reactions assessed by OJIP kinetics in three *brassica* crops after drought and recovery. Appl Sci.

[34] Xu S, Liu Z, Han S, Chen Z, He X, Zhao H (2023). Exploring the sensitivity of solar-induced chlorophyll fluorescence at different wavelengths in response to drought. Remote Sens.

[35] Maxwell K, Johnson GN (2000). Chlorophyll fluorescence-a practical guide. J Exp Bot.

[36] Oxborough K (2004). Imaging of chlorophyll *a* fluorescence: theoretical and practical aspects of an emerging technique for the monitoring of photosynthetic performance. J Exp Bot.

[37] Zhou R, Yu X, Kjær KH, Rosenqvist E, Ottosen CO, Wu Z (2015). Screening and validation of tomato genotypes under heat stress using *Fv/Fm* to reveal the physiological mechanism of heat tolerance. Environ Exp Bot.

[38] Saddiq MS, Iqbal S, Hafeez MB, Ibrahim AMH, Raza A, Fatima EM (2021). Effect of salinity stress on physiological changes in winter and spring wheat. Agronomy.

[39] Zou J, Hu W, Li YX, He JQ, Zhu HH, Zhou ZG (2020). Screening of drought resistance indices and evaluation of drought resistance in cotton (*Gossypium hirsutum* L). J Integr Agric.

[40] González-Morales SI, Chávez-Montes RA, Hayano-Kanashiro C, Alejo-Jacuinde G, Rico-Cambron TY, De Folter S et al. Regulatory network analysis reveals novel regulators of seed desiccation tolerance in *Arabidopsis thaliana*. Proc. Natl. Acad. Sci. U.S.A. 2016;113:E5232–41. 10.1073/pnas.1610985113 PMC5024642 27551092

[41] Wu Y, Deng Z, Lai J, Zhang Y, Yang C, Yin B (2009). Dual function of *Arabidopsis ATAF1* in abiotic and biotic stress responses. Cell Res.

[42] Han YJ, Kim YM, Hwang OJ, Kim JI (2015). Characterization of a small constitutive promoter from *Arabidopsis* translationally controlled Tumor protein (*AtTCTP*) gene for plant transformation. Plant Cell Rep.

[43] Engler C, Youles M, Gruetzner R, Ehnert TM, Werner S, Jones JDG (2014). A Golden Gate modular cloning toolbox for plants. ACS Synth Biol.

[44] Marillonnet S, Werner S, Castilho A (2015). Assembly of multigene constructs using golden gate cloning. Glyco-Engineering: methods and protocols.

[45] Martinez-Trujillo M, Limones-Briones V, Cabrera-Ponce JL, Herrera-Estrella L (2004). Improving transformation efficiency of *Arabidopsis thaliana* by modifying the floral dip method. Plant Mol Biol Rep.

[46] Fernandez-Jaramillo AA, Duarte-Galvan C, Contreras-Medina LM, Torres-Pacheco I, Romero-Troncoso R, de J, Guevara-Gonzalez RG (2012). Instrumentation in developing chlorophyll fluorescence biosensing: a review. Sensors.

[47] Aissaoui F, Chehab H, Bader B, Salem AB, M’barki N, Laamari S (2016). Early water stress detection on olive trees (*Olea europaea* L. cvs ‘chemlali’ and ‘Chetoui’) using the leaf patch clamp pressure probe. Comput Electron Agric.

[48] Cahyo AN, Murti RH, Putra ETS, Nuringtyas TR, Fabre D, Montoro P (2021). Assessment of factual measurement times for chlorophyll-*a* fluorescence in rubber (*Hevea brasiliensis*) clones. Biodiversitas.

[49] Chen D, Yuan K, Zhang J, Wang Z, Sun Z, Zhang H (2022). Response analysis of fluorescence parameters of tomato seedlings oriented to vertical light environment adaptation. Plant Sci.

[50] Chen X, Yuan H, Chen R, Zhu L, He G (2003). Biochemical and photochemical changes in response to triacontanol in rice (*Oryza sativa* L). Plant Growth Regul.

[51] Thoren D, Thoren P, Schmidhalter U (2010). Influence of ambient light and temperature on laser-induced chlorophyll fluorescence measurements. Europ J Agronomy.

[52] Mantova M, Herbette S, Cochard H, Torres-Ruiz JM (2022). Hydraulic failure and tree mortality: from correlation to causation. Trends Plant Sci.

[53] Fleming A (2006). Metabolic aspects of organogenesis in the shoot apical meristem. J Exp Bot.

[54] Munné-Bosch S, Alegre L (2004). Die and let live: Leaf senescence contributes to plant survival under drought stress. Funct. Plant Biol.

[55] Bechtold U, Lawson T, Mejia-Carranza J, Meyer RC, Brown IR, Altmann T (2010). Constitutive salicylic acid defences do not compromise seed yield, drought tolerance and water productivity in the *Arabidopsis* accession C24. Plant Cell Environ.

