# Supplementary material for: A non-invasive method to predict drought survival in Arabidopsis using quantum yield under light conditions
Source: Plant Methods. 2023 Nov 15;19:127. doi: 10.1186/s13007-023-01107-w (PMC10647164; doi:10.1186/s13007-023-01107-w)
Supplement: Supplementary file 1 — Additional file 1: Supplementary Table S1: Primers used in this study. Supplementary Table S2: Golden Gate MoClo Vectors used in this study [file 13007_2023_1107_MOESM1_ESM.pdf]

Supplementary Table S1: Primers used in this study.

Name	Gene ID		Primer sequence
**TCTP promoter**	AT3G16640	Fw	ttgaagacaaggagCCAACACTCGAATCCCCAC
		Rv	ttgaagacaacattGGTCGCTTATTGATTGTTTTCTCT
**ATAF1 CDS**	AT1G01720	Fw	ttgaagacaaaATGTCAGAATTATTACAGTTGCCTC
		Rv	ttgaagacaaaagcCTAGTAAGGCTTCTGCATGTACA
**ATAF1 RT-qPCR**	AT1G01720	Fw	GAGCTTCCTGGTTTAGCCTTGT
		Rv	AACCCAATCATCCAGCCTGAG
**PLATZ1 RT-qPCR**	AT1G21000	Fw	TCCTCAATGAAAGACCTCAGCC
		Rv	TGGGACCGTAGTCATCGAATCTAA
		Rv (cDNA)	CTTTGATCACCATTGGGACCGT
**ACTIN2 RT-qPCR**	AT3G18780	Fw	GAATTGTCTCGTTGTCCTCCTCAC
		Rv	TGCAAATCCAGCCTTCACCATAC

Supplementary Table S2: Golden Gate MoClo Vectors used in this study (43).

Kit	Vector Name	Description
MoClo Plant Tool Kit	pICH41295	Level 0 acceptor for promoter + 5UTR
	pICH41308	Level 0 acceptor for coding sequence
	pICH47732	Level 1 acceptor for position 1 modules in forward orientation
	pICH47742	Level 1 acceptor for position 2 modules in forward orientation
	pAGM4673	Level 2 acceptor
	pICSL70005	End-link 2 with lacZ acceptor site
Golden Gate Plant Parts Kit	pICH41432	OCS terminator (*A. tumefaciens*)
	pICSL70005	Bialaphos resistance cassette
