# Supplementary material for: A non-invasive method to predict drought survival in Arabidopsis using quantum yield under light conditions
Source: Plant Methods. 2023 Nov 15;19:127. doi: 10.1186/s13007-023-01107-w (PMC10647164; doi:10.1186/s13007-023-01107-w)
Supplement: Supplementary file 2 — Additional file 2: Supplementary Figure S1: Relative expression and drought survival analysis of two Arabidopsis transgenic lines. Supplementary Figure S2: Plants of different Arabidopsis genotypes grown under well-watered conditions [file 13007_2023_1107_MOESM2_ESM.pdf]

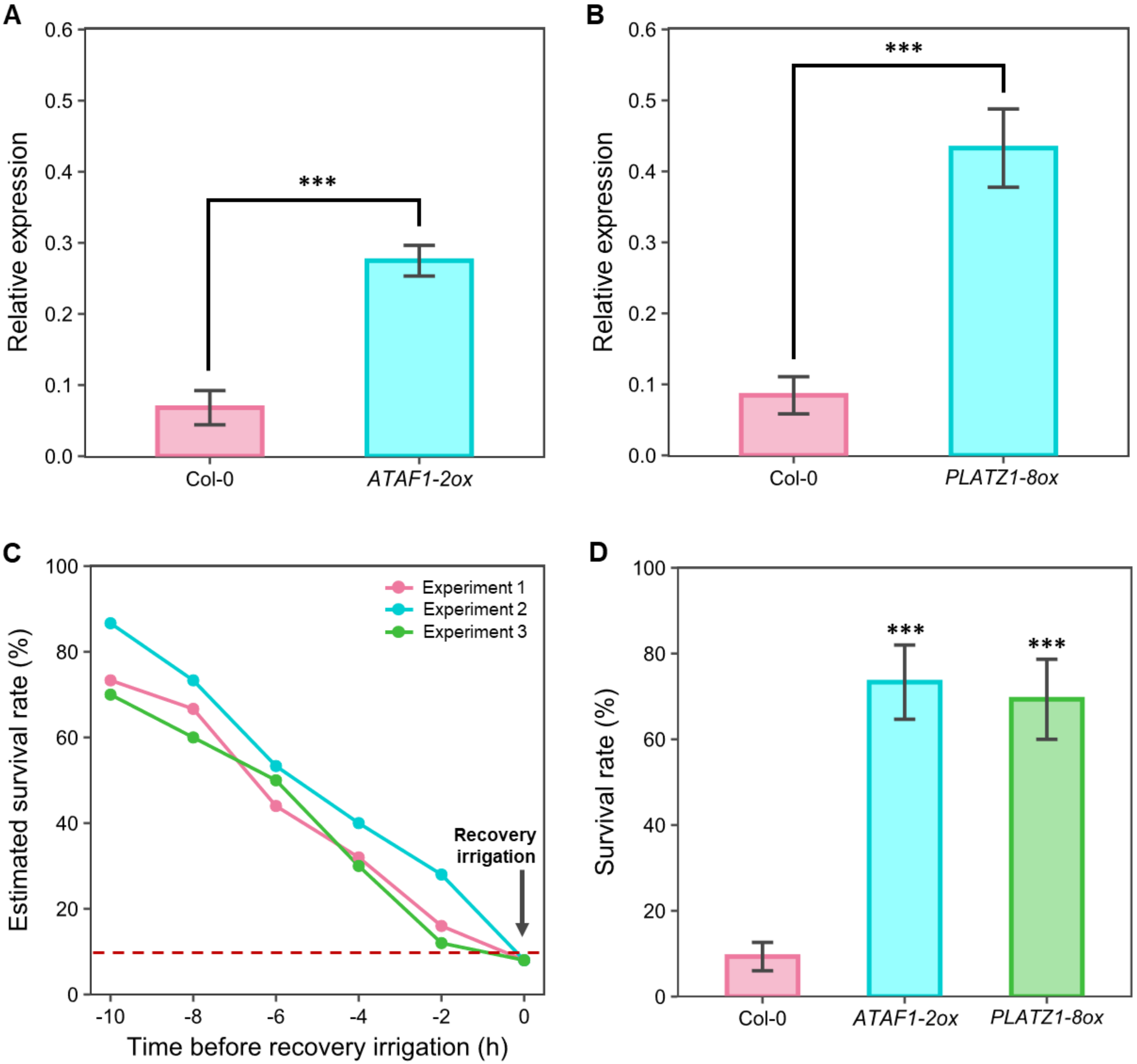

Supplementary Figure S1: Relative expression and drought survival analysis of two Arabidopsis transgenic lines. A) and B) Analysis of *ATAF1* (A) and *PLATZ1* (B) expression level in their respective transgenic lines and Col-0 by real-time PCR. *ACT2* was used as reference gene. Bar plots show the mean and standard deviation of three biological and technical replicates. Asterisks, significant differences (Student's t-test, P<0.001). C) Estimation of the survival rate of Col-0 at different hours before recovery irrigation based on values of Fv'/Fm' from each Col-0 plant. Line plot shows the individual estimation of three independent experiments (5 biological replicates per experiment, n=25 plants). Red line, maximum survival rate expected for Col-0; arrow, recovery irrigation supplying. D) Survival rate of the different genotypes after receiving a recovery irrigation. Bar plot shows the mean and standard deviation of 15 biological replicates in three independent experiments (n=75 plants). Asterisks, significant differences (Fisher's exact test, P<0.001).

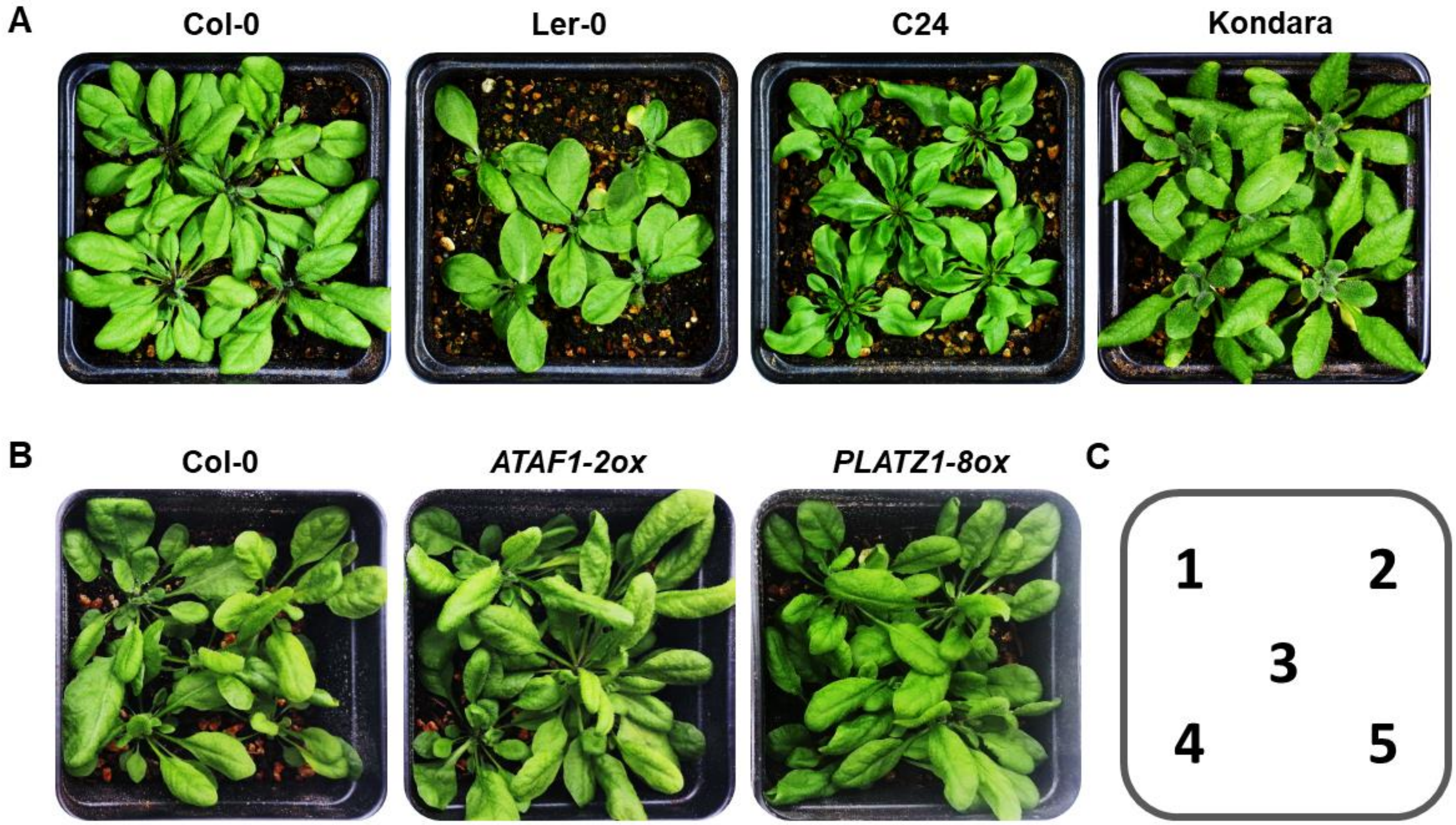

Supplementary Figure S2: Plants of different Arabidopsis genotypes grown under well-watered conditions. Representative rosettes of A) Arabidopsis accessions and B) *ATAF1-2ox* and *PLATZ1-8ox* transgenic lines grown for 40 days in the control treatments. C)Plant distribution in pots followed in all the experiments here reported.
