# Supplementary material for: A non-invasive method to predict drought survival in Arabidopsis using quantum yield under light conditions
Source: Plant Methods. 2023 Nov 15;19:127. doi: 10.1186/s13007-023-01107-w (PMC10647164; doi:10.1186/s13007-023-01107-w)
Supplement: Supplementary file 3 — Additional file 3: Detailed statistical validation of the method [file 13007_2023_1107_MOESM3_ESM.pdf]

Assessing the robustness of models for predicting plant survival under water stress as function of quantum yield
SUPPLEMENTARY TO
"*A non-invasive method to predict drought survival in arabidopsis using quantum yield under light conditions*"

OCTAVIO MARTÍNEZ.

Evaluating the predictive capability of a statistical model for forecasting future observations is crucial to ensure its usefulness in a specific situation. The main text of the paper proposes the use of a non-invasive method to estimate the survival probability of *Arabidopsis thaliana* plants after severe drought treatments. Specifically, by utilizing different *Arabidopsis* ecotypes (Col-0, Ler-0, C24, and Kondara), a significant and high correlation was discovered between plant survival and the quantum yield (QY), which we represent here as the parameter `FVFM`.

The purpose of this statistical addendum is to assess the predictive capability of statistical models in determining the plants' survival phenotype. This evaluation employs a model that excludes the observations it attempts to predict.

All statistical calculations were performed in R (R Core Team, 2013) version 4.2.2 running under macOS Ventura 13.5.1, and data as well as log files with calculations and results are available upon request. Summaries of results are presented as boxes of text, copied from the R command window.

Experiments and Data

The experiments analyzed in this section correspond to the ones mentioned as "*Calibration Stage*" experiments, and were designed to evaluate plant survival as function of the FVFM parameter. The experimental unit were individual plants of each one of the 4 ecotypes (Col-0, Ler-0, C24, and Kondara), grown in pots with 5 plants and with 4 biological replicates per experiment. All plants were subjected to the same drought treatment, and FVFM measurements began when the plants showed severe dehydration symptoms. Pots were watered at different times to obtain FVFM values in the rank between 0.00 and 0.77 (normal conditions). Plant survival was evaluated seven days after taking the FVFM measurement. The whole experiment was repeated independently 3 times, to obtain a total of 60 evaluations of FVFM and plant survival for each one of the 4 ecotypes, thus we have a total of $60 \times 4 = 240$ measurements.

`Box 1` shows a general description and initial analysis of the data.

```
------------------------------------------------------------------------------------
# Box 1. Data summaries and initial analysis.
# Data summary (object "thelma")
> dim(thelma)
[1] 240   4
> names(thelma)
[1] "Trat"      "FVFM"      "Phenotype" "NumPhe"
> table(thelma$Trat) # The different Arabidopsis ecotypes
  a.Col-0   b.Ler-0     c.C24 d.Kondara
       60        60        60        60
> table(thelma$Phenotype) # The different Arabidopsis phenotype
```

Date: September 10, 2023.

```
a.Survived     b.Died
       120        120
> table(thelma$Phenotype, thelma$Trat)
             a.Col-0 b.Ler-0 c.C24 d.Kondara
  a.Survived      30      30    30        30
  b.Died          30      30    30        30
> summary(thelma$FVFM) # Parameter measured in each experimental unit
   Min. 1st Qu.  Median    Mean 3rd Qu.    Max.
 0.0300  0.2475  0.3650  0.3699  0.5000  0.7000
> tapply(thelma$FVFM, thelma$Trat, mean)
  a.Col-0   b.Ler-0     c.C24 d.Kondara
0.4136667 0.3461667 0.3445000 0.3753333

# Performing an ANOVA to see if the measures of FVFM are dependent on the
# genotypes (Treat) and Phenotype (survival of the plants)
> summary(aov(FVFM ~ Trat*Phenotype, data=thelma))
                Df Sum Sq Mean Sq F value   Pr(>F)
Trat             3  0.189   0.063   7.375 9.67e-05 ***
Phenotype        1  3.866   3.866 452.062  < 2e-16 ***
Trat:Phenotype   3  0.007   0.002   0.262    0.853
Residuals      232  1.984   0.009
-----------------------------------------------------------------------------------------
```

In `Box 1` we can see that the values of FVFM are significantly different for both, the ecotypes (“`Trat`”) and surviving phenotype (“`Phenotype`”), however there is no interaction between those two sources of variation. Given this it makes sense to perform individual statistical analyses for each one of the four *Arabidopsis* ecotypes separately.

Analysis

A Generalized Linear Model (GLM) for the surviving genotype as function of FVFM in the Col-2 ecotype. Having observed that the surviving phenotype has a strong relation with FVFM within each genotype, we can estimate a GLM for the surviving probability as function of the observed FVFM parameter, assuming the Binomial family distribution (see, for example, Draper and Smith (2014)).

Box 2 presents the R calculations and output of the model for the probability of surviving given the value of FVFM in the Col-0 ecotype.

```
-----------------------------------------------------------------------------------------
# Box 2. a GLM for the surviving probability as function of FVFM.
# Isolate the data for the Columbia 0 ecotype
> th.Col <- thelma[thelma$Trat=="a.Col-0", c(4, 2)]

# Estimate the GLM for the numeric phenotype
# (NumPhe = 1 when the plant survived and 0 otherwise)
# as function of FVFM, assuming the Binomial distribution
# in the Col-0 ecotype.
> mod.th.Col <- glm(NumPhe ~ FVFM, data=th.Col, family = binomial)
> summary(aov(mod.th.Col))
            Df Sum Sq Mean Sq F value   Pr(>F)
FVFM         1  9.421   9.421   97.96 4.54e-14 ***
Residuals   58  5.579   0.096
> summary(mod.th.Col)
```

```
Coefficients:
            Estimate Std. Error z value Pr(>|z|)
(Intercept)  -21.041      7.371  -2.855  0.00431 **
FVFM          46.730     15.910   2.937  0.00331 **

# Obtain the predicted probabilities for each value of FVFM
> th.Col$p.est <- predict(mod.th.Col, type="response")

# Confirming: p.est is much lower when the numeric phenotype is 0 (plant died)
> tapply(th.Col$p.est, th.Col$Pphe, mean)
          0           1
0.002999883 0.787736910

# Calculate the predicted phenotype, Pphe, taking as threshold an estimated probability of 1/2:
> th.Col$Pphe <- 1*(th.Col$p.est >= 0.5)
> table(th.Col$NumPhe, th.Col$Pphe)
     0  1
  0 27  3
  1  2 28
# Changing attributes to reflect categories
> tab1 <-table(th.Col$NumPhe, th.Col$Pphe)
> attributes(tab1)$dimnames <- list(c("Dead", "Alive"), c("P.Dead", "P.Alive"))
> tab1
      P.Dead P.Alive
Dead      27       3
Alive      2      28
# "tab1" is presented as Table 1.

# Plots
# Figure 1: Estimated survival probability as function of FVFM.
plot(th.Col$FVFM, th.Col$p.est, xlab="FVFM", ylab="Estimated probability of survival")
grid()
abline(h=0.5, col="red")

# Figure 2: Box plot of Estimated survival probability by survival phenotype.
boxplot(p.est ~ NumPhe, data=th.Col,
     xlab="Numeric phenotype (0 plant died, 1 plant survived)",
     ylab="Estimated probability of survival")
abline(h=0.5, col="red")
grid(nx=NA, ny=NULL)
text(c(1, 2), tapply(th.Col$p.est, th.Col$Pphe, mean), label="*", col="blue", cex=3)
---------------------------------------------------------------------------------------
```

In Box 2 we see that the GLM estimated for the probability of survival as function of the FVFM parameter is highly significant (p-value $= 4.54 \times 10^{-14}$). Figures 1 and 2 show graphics of the results.

In Figure 1 we can observe the strong dependence that exist between the estimated probability of survival and the FVFM in the Col-0 ecotype. In this plot we can see that FVFM $= 0.45$ (vertical blue line) is a threshold for the estimated probability of survival, i.e., all points below such threshold have an estimated probability of survival less than 0.5, while all points above that threshold have an estimated probability of survival less larger than 0.5.

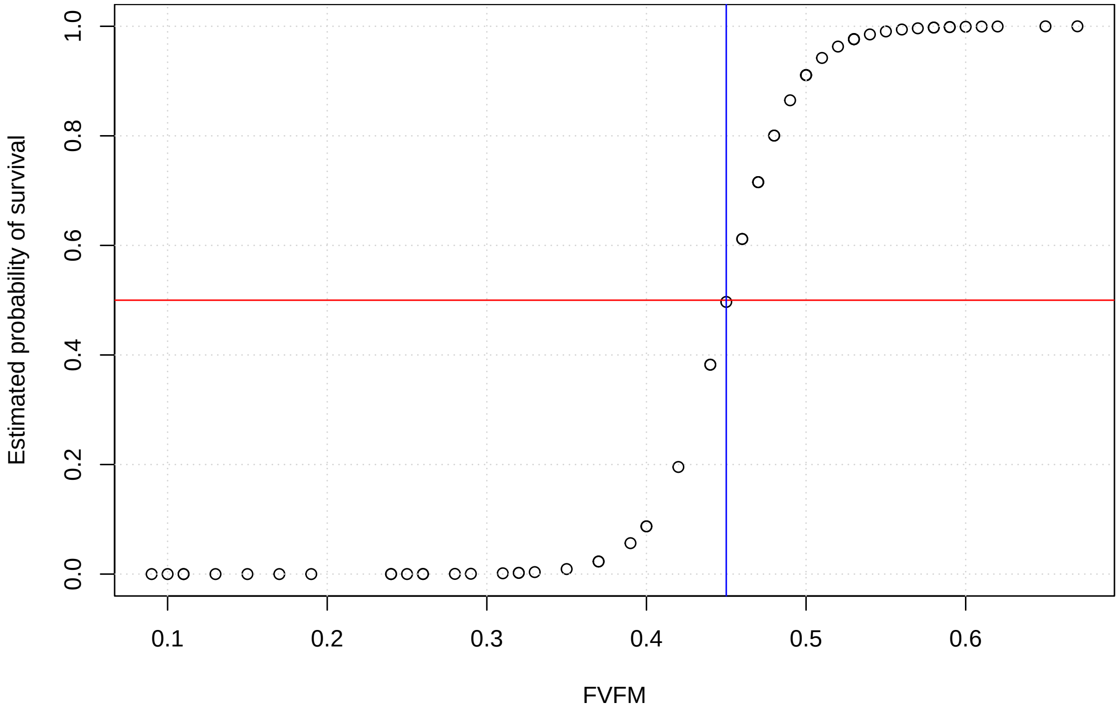

FIGURE 1. **Estimated probability of plant survival as function of the FVFM parameter in the Col-2 ecotype.**

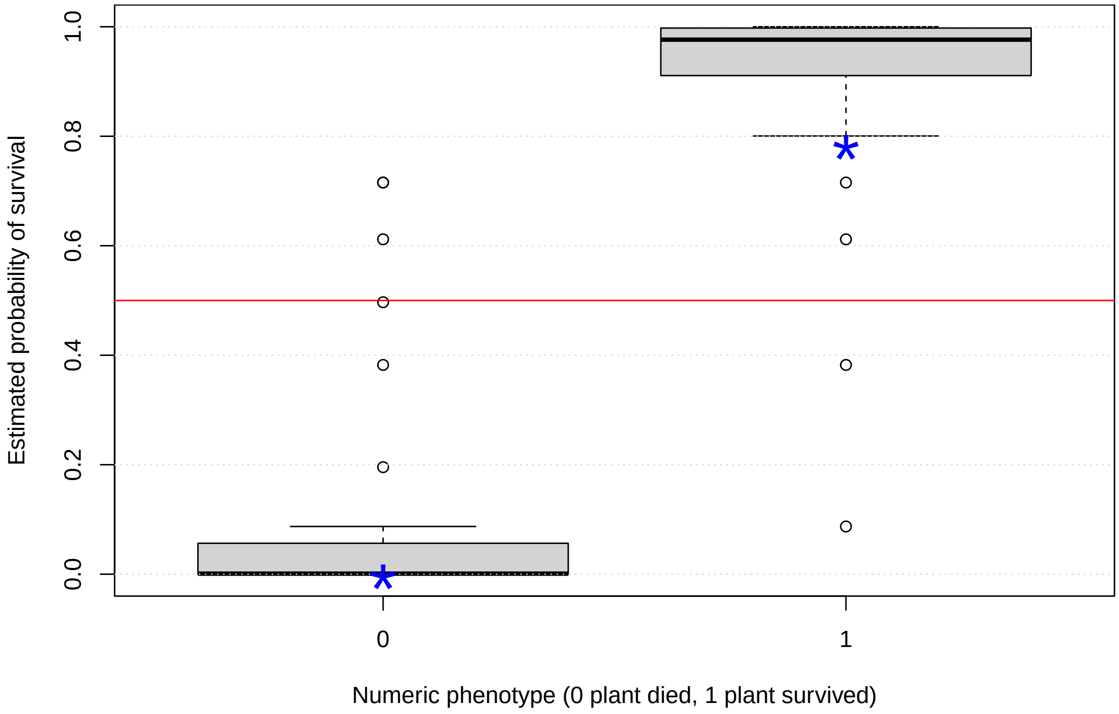

FIGURE 2. **Box plots for the estimated probability of survival group by numeric phenotype.**

On the other hand, Figure 2 shows the box plot of the distributions of the estimated probability of survival in the two phenotypes, plants that died (numeric phenotype = 0) and plants that survived (numeric phenotype = 1), in both cases 7 days after taking the FVFM measurement.

To predict the state (phenotype) of a plant after the seven days after taking the FVFM value we can use the criterion "*predict that a plant will survive after seven days if the estimated probability of survival is equal of grater than 1/2*". Table 1 presents the contingency table showing the numbers of plants in each state in rows and the state predicted by the model in columns (object "`tab1`" in `Box 2`).

TABLE 1. True (rows) and Predicted (columns) states of the plants.

True State:	Predicted to be Dead	Alive
Dead	27	3
Alive	2	28

From Table 1 we can see that the proportion of correct predictions of the model is $27 + 28 = 55$, i.e., the model using all 60 observations correctly predicted the state of the plant in $\approx 92\%(55/60 \approx 0.9167)$ of the cases, indicating that the FVFM parameter is highly efficient to predict survival in the conditions of the experiment. Also from Table 1 we can see that the model erroneously predicts in 3 cases that the plants will survive when in fact after 7 days they are found to be dead; this implies that the model is giving a rate of false positives of $3/60 = 0.05$ These three cases correspond to the three points found over or above the 0.5 threshold in the left hand side box plot (numeric phenotype 0) in Figure 2. On the other hand, the model gives 2 false negatives, i.e., in two cases it predicted that the plants will be dead when in fact they are found to be alive after 7 days; this implies a rate of false negatives of $2/60 \approx 0.0333$. Those two cases correspond to the points found below the 0.5 threshold in the right hand side box plot (numeric phenotype 1) in Figure 2.

Assessing the power of the method to predict the phenotype. To evaluate how good is the method employed to predict plant status as function of the FVFM parameter, we must split the data into two almost independent subsets, say, a training set from which the parameters of the model are estimated, and the other (not employed in the estimation process) where we could test the predictions. We have 60 observations available per genotype and ideally, both, the training and the validation sets could be of the same size ($n = 30$). However, in this case the process to estimate the GLM fails to converge when the number of data points is less than approximately 50 (data not shown). Thus, to obtain a training set we will select at random 50 of the 60 data points, and using the estimated parameters in the remaining 10 plants to test the power of the method. Because the "training" and "validation" sets are almost independent, i.e., they can be considered to be the results of independent experiments performed under the same conditions each time, the power of the method to predict the phenotype as function of the FVFM parameter will be unbiasedly estimated.

By repeating the random selection of the "training" ($n = 50$) and "validation" ($n = 10$) sets many times, say $B = 1000$, we obtain in each case a good measure of the expected performance of the method to predict future observations (Harrell and Harrell, 2001). `Box 3` presents the calculations and general results of this procedure with the data of the Col-0 ecotype.

```
-----------------------------------------------------------------------------------------
# Box 3. Obtaining 1000 estimates of training and validation in the Col-0 data.frame.
# Define a data.frame to allocate each result.
> samp.th.Col <- data.frame(In=rep(NA, 1000), InSE=NA, InZ=NA, InPz=NA,
Be=NA, BeSE=NA, BeZ=NA, BePz=NA, N0P0=NA, N1P0=NA, N0P1=NA, N1P1=NA)

# Content of each variable:
# "In"  Estimated intercept of the model.
```

```
# "InSE" Estimated standard error for the intercept of the model.
# "InZ" Estimated z-value for testing In==0.
# "InPz" P value for the test In==0.
# "Be"  Estimated  coefficient of the model.
# "BeSE" Estimated standard error for the  coefficient of the model.
# "BeZ" Estimated z-value for testing Be==0.
# "BePz" P value for the test Be==0.
# "N0P0" True positives (Dead predicted to be dead)
# "N1P0" False positives (Alive predicted to be dead)
# "N0P1" False negatives (Dead predicted to be alive)
# "N1P1" True negative (Alive predicted to be alive)

# Obtain the results
# Initialize the random seed to make results replicable
set.seed(1959)
j <- 0
for(i in 1:1100){
# Select a random set for training (estimation)
temp.i <- sort(sample(c(1:60), size=50))
th.Col.tra <- th.Col[temp.i,1:2] # Dataset for estimation (training)
th.Col.pre <- th.Col[setdiff(c(1:60), temp.i),1:2] # Dataset for prediction (test)
# Estimate the model
temp.mod <- glm(NumPhe ~ FVFM, data=th.Col.tra, family = binomial)
if(temp.mod$converged == TRUE) {
j=j+1
samp.th.Col[j, 1:4] <- summary(temp.mod)$coef[1,]
samp.th.Col[j, 5:8] <- summary(temp.mod)$coef[2,]
# Predict the results for the cases NOT used in training
temp.pre <- predict(temp.mod, newdata=th.Col.pre, type="response")
th.Col.pre$p.pred <- temp.pre
th.Col.pre$PreFen <- 1*(th.Col.pre$p.pre >= 0.5)
table(th.Col.pre$NumPhe, th.Col.pre$PreFen)
samp.th.Col[j, 9:12] <- as.vector(table(th.Col.pre$NumPhe, th.Col.pre$PreFen))
}
}
# Note: there are some warnings and non-convergences, at the end we get a total
# of 1000 evaluations:
> nrow(samp.th.Col)
[1] 1000
> summary(samp.th.Col)
       In               InSE             InZ               InPz
 Min.   :-80.33   Min.   : 6.405   Min.   :-2.861   Min.   :0.004227
 1st Qu.:-22.02   1st Qu.: 7.160   1st Qu.:-2.726   1st Qu.:0.006404
 Median :-20.09   Median : 7.462   Median :-2.654   Median :0.007953
 Mean   :-23.42   Mean   : 9.588   Mean   :-2.571   Mean   :0.013149
 3rd Qu.:-19.31   3rd Qu.: 8.587   3rd Qu.:-2.545   3rd Qu.:0.010915
 Max.   :-17.34   Max.   :48.989   Max.   :-1.534   Max.   :0.125066
       Be              BeSE             BeZ              BePz
 Min.   : 38.96   Min.   : 13.91   Min.   :1.541   Min.   :0.003041
 1st Qu.: 42.99   1st Qu.: 15.47   1st Qu.:2.627   1st Qu.:0.005074
 Median : 44.83   Median : 16.18   Median :2.734   Median :0.006265
 Mean   : 51.83   Mean   : 20.70   Mean   :2.641   Mean   :0.011432
```

```
 3rd Qu.: 48.74   3rd Qu.: 18.49   3rd Qu.:2.802   3rd Qu.:0.008620
 Max.   :172.91   Max.   :105.15   Max.   :2.964   Max.   :0.123428
      N0P0            N1P0            N0P1            N1P1
 Min.   :1.000   Min.   :0.000   Min.   :0.000   Min.   :1.000
 1st Qu.:3.000   1st Qu.:0.000   1st Qu.:0.000   1st Qu.:4.000
 Median :4.000   Median :0.000   Median :1.000   Median :5.000
 Mean   :4.305   Mean   :0.379   Mean   :0.653   Mean   :4.693
 3rd Qu.:5.000   3rd Qu.:1.000   3rd Qu.:1.000   3rd Qu.:6.000
 Max.   :8.000   Max.   :9.000   Max.   :4.000   Max.   :9.000
-----------------------------------------------------------------------------------------
```

When using the full set of 60 data points, we obtained estimated values of the parameters that were $\hat{a} = -21.04$ for the intercept and $\hat{b} = 46.73$ for the slope, and in both cases the parameters were significantly different from zero, with p values 0.004 and 0.003, respectively (see `Box 2`). From `Box 3` we can notice that the medians of the $B = 1000$ replicates of the training model using in each case 50 random data points of the 60 ones available (83%) gives a median of -20.09 for the intercept and 44.83 for the slope, with median p values of 0.008 and 0.006, respectively. This means that the resampling procedure using only 83% of the data gave models close to the ones obtained with the full data set and with only a relatively small decrease of significance.

Now we need to analyze the rate of correct predictions obtained from each one of the $B = 1000$ training models in the ten independent data used for validation in each case. `Box 4` presents the rearrangement and plotting of the results of the resampling procedure.

```
-----------------------------------------------------------------------------------------
# Box 4 -  Rearrangement and plotting of the results in object samp.th.Col
> sum.samp.th.Col <- data.frame(fC=(samp.th.Col$N0P0+samp.th.Col$N1P1)/10,
     fTP=samp.th.Col$N0P0/10, fFP=samp.th.Col$N1P0/10,
     fFN=samp.th.Col$N0P1/10, fTN=samp.th.Col$N1P1/10)

# Making a data.frame to plot the results
> res.samp.th.Col <- data.frame(group=rep(c("Correct", "True Positive",
     "False Positive", "False Negative", "True Negative"), each=1000),
     value=c(sum.samp.th.Col$fC, sum.samp.th.Col$fTP,
     sum.samp.th.Col$fFP, sum.samp.th.Col$fFN, sum.samp.th.Col$fTN))

> tapply(res.samp.th.Col$value, res.samp.th.Col$group, mean)
       Correct False Negative False Positive  True Negative  True Positive
        0.8998         0.0653         0.0379         0.4693         0.4305

> apply(sum.samp.th.Col, 2, median)
 fC fTP fFP fFN fTN
0.9 0.4 0.0 0.1 0.5

### Figure 3
boxplot(value ~ group, data=res.samp.th.Col, cex.axis=0.85,
     col=c("green3", "red2", "red2", "green3", "green3"),
     xlab="Group of classified predictions",
     ylab="Relative frequency in 1000 replicates")
grid(nx=NA, ny=NULL)
text(c(1:5), tapply(res.samp.th.Col$value, res.samp.th.Col$group, mean),
     labels="*", cex=3, col="white")
text(c(1:5)+.25, c((27+28)/60, 2/60, 3/60, 28/60, 27/60),
```

```
    labels="X", font=2, cex=1, col="white")
------------------------------------------------------------------------------
```

Figure 3 presents box plots for the relative frequencies of the four groups of the predictions classified as "Correct", "True Positive", "False Positive", "False Negative" and "True Negative" which were obtained by $B = 1000$ iterations of the validation process (see `Box 4`).

Note that in each training / validation case the results of each one of the 10 predictions could be classified as "Correct" if the model correctly classified the phenotype; see Table 1 for the results in the original model which employed all the 60 data points. See also that table for the definitions of the other four categories: "True Positive", "False Positive", "False Negative" and "True Negative".

Figure 3 summarizes the validation results by showing the box plot distribution of the relative frequencies of each one of the groups of in the $B = 1000$ random replications of the process.

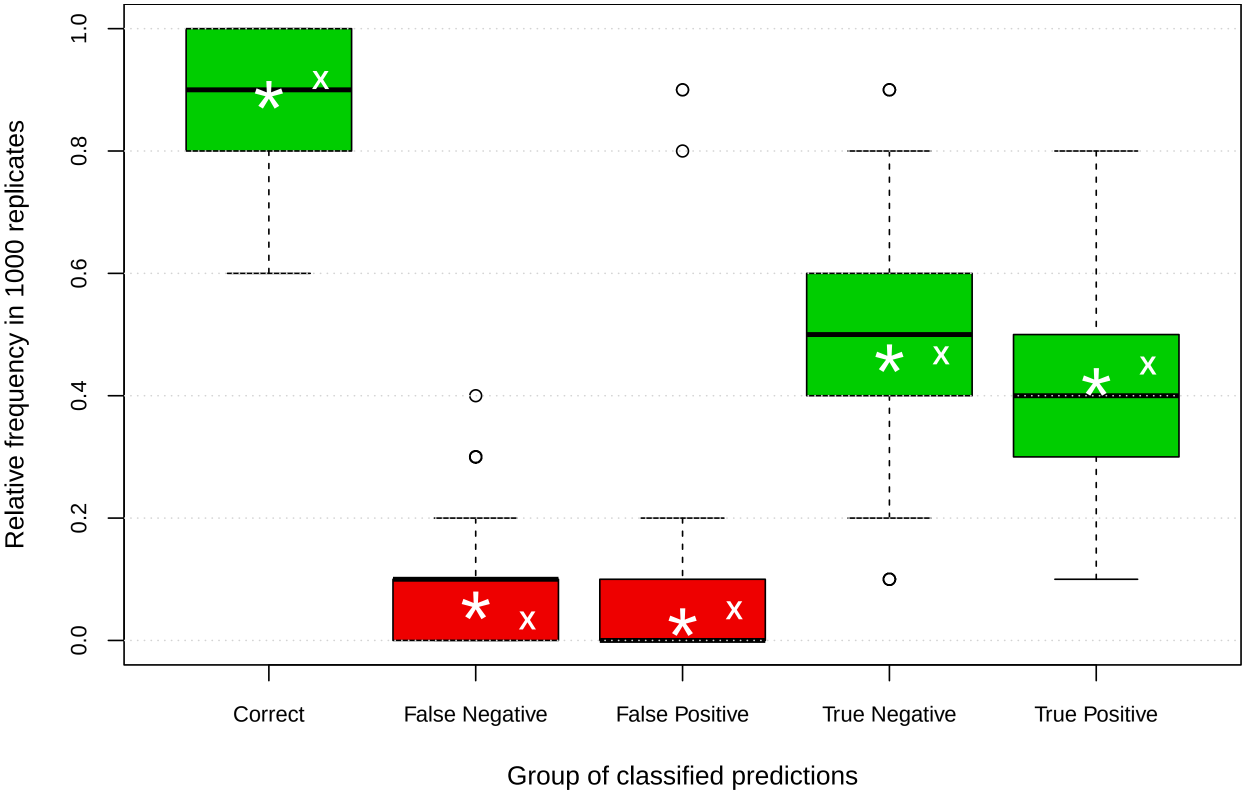

FIGURE 3. **Box plots for the validation of the procedure in the Col-0 ecotype.** White asterisks show the mean of each frequency, while white "X" show the estimates in the model with all 60 data points (see `Box 4`).

In Figure 3 we can observe how the results of the validation process are statistically congruent with the results obtained when using all 60 data points in the original model. First, in all 5 groups, the values estimated from the full model (60 data points) and shown as white "X" in the figure, are close to the mean and median values (white asterisks and bold black lines respectively). This means that in the long run the prediction of new data from models using only part of the data result both, accurate and precise enough to adopt the method proposed in the paper to predict the phenotype of interest.

For example, in the box plot for the "Correct" group in Figure 3 we see that the proportion of correct results in the full model was of 92%, while the mean of the $B = 1000$ replicates gave correct results for data not employed in the training in 90% of the cases, i.e., only 2% less in average that the model using the whole data set. Furthermore, the interquartile range in the "Correct" group goes from 0.8 to 1 with a median of 0.9, suggesting that the correct prediction of future phenotypes (dead or alive) using

the FVFM measurement has high probability of success. On the other hand, the rates of the 4 groups that segregate the results, i.e., the sets of "False Negative", "False Positive", "True Negative" and "True Positive" shown in Figure reff3 are also close to the values estimated from the full model, demonstrating the statistical robustness of the method.

It could be argued that in the paper the selection of the FVFM thresholds employed are heuristically based in models simpler than the GLM employed here; i.e., the suitable FVFM threshold was based in the estimation of the FVFM value that gave the best partition between phenotypes observed *a posteriori*. However, this do not alter the fact that FVFM are good *a priori* predictor of the phenotype, and both, simple graphical models as well as the GLM will give concordant results. As shown in Figure 1 of this report, the FVFM threshold to determine the sharp change in surviving probability is alike the thresholds employed in the figures of the paper because determining the empirical threshold is almost equivalent to the fitting of the GLM.

Estimation and validation in other ecotypes

The calibration experiments were performed for 4 ecotypes: Col-0, Ler-0, C24, and Kondara. Previously, we have seen that ecotype (source "`Trat`" in the ANOVA of `Box 1`) as well as the survival phenotype (source "`Phenotype`" in the ANOVA of `Box 1`) have strong and significant effect on the FVFM parameter.

We have examined in detail the results for the Col-0 ecotype (Figures 1 and 2), corroborating that the FVFM parameter has plenty of statistical information to predict the survival phenotype with accuracy and precision in the Col-0 ecotype (Figure 3). In this section we present the results for the other 3 ecotypes, Ler-0, C24, and Kondara. Analysis in R were performed following exactly the same pipeline than with the Co.-0 ecotype, and thus boxes with the R calculations for each one of these 3 ecotypes are omitted for brevity, but the R objects and log files are available upon request.

Table 2 presents the estimated of the parameters for the GLM in the four ecotypes, while Figure 4 presents the estimated probabilities for those models as function of the FVFM parameter also by ecotype.

Table 2. Parameters (intercepts and slopes) estimated for the GLM in the four ecotypes.

	Intercept			
Ecotype	Estimate	Std. Error	z-value	p-value
Col-0	-21.04	7.37	-2.85	0.00431
Ler-0	-12.45	3.89	-3.20	0.00138
C24	-16.36	6.08	-2.69	0.00708
Kondara	-18.54	6.77	-2.74	0.00615
	Slope (on FVFM)			
Ecotype	Estimate	Std. Error	z-value	p-value
Col-0	46.73	15.91	2.94	0.00331
Ler-0	37.58	11.61	3.24	0.00120
C24	48.42	18.01	2.69	0.00719
Kondara	51.16	19.20	2.67	0.00770

In Table 2 we can see that the estimated parameters of the GLM (both, intercept and slope) depend on the ecotype, a fact previously seen in `Box 1`. Nevertheless, in all four ecotypes the statistical tests performed for the null hypotheses in these estimates are always highly significant, presenting p-values < 0.008 in all cases. This implies that, in general, the FVFM parameter has a definite significant influence over the surviving phenotype.

The differences in the fitted models for the 4 ecotypes can be appreciated in Figure 4, which presents the estimated probabilities as functions of the FVFM parameter per ecotype.

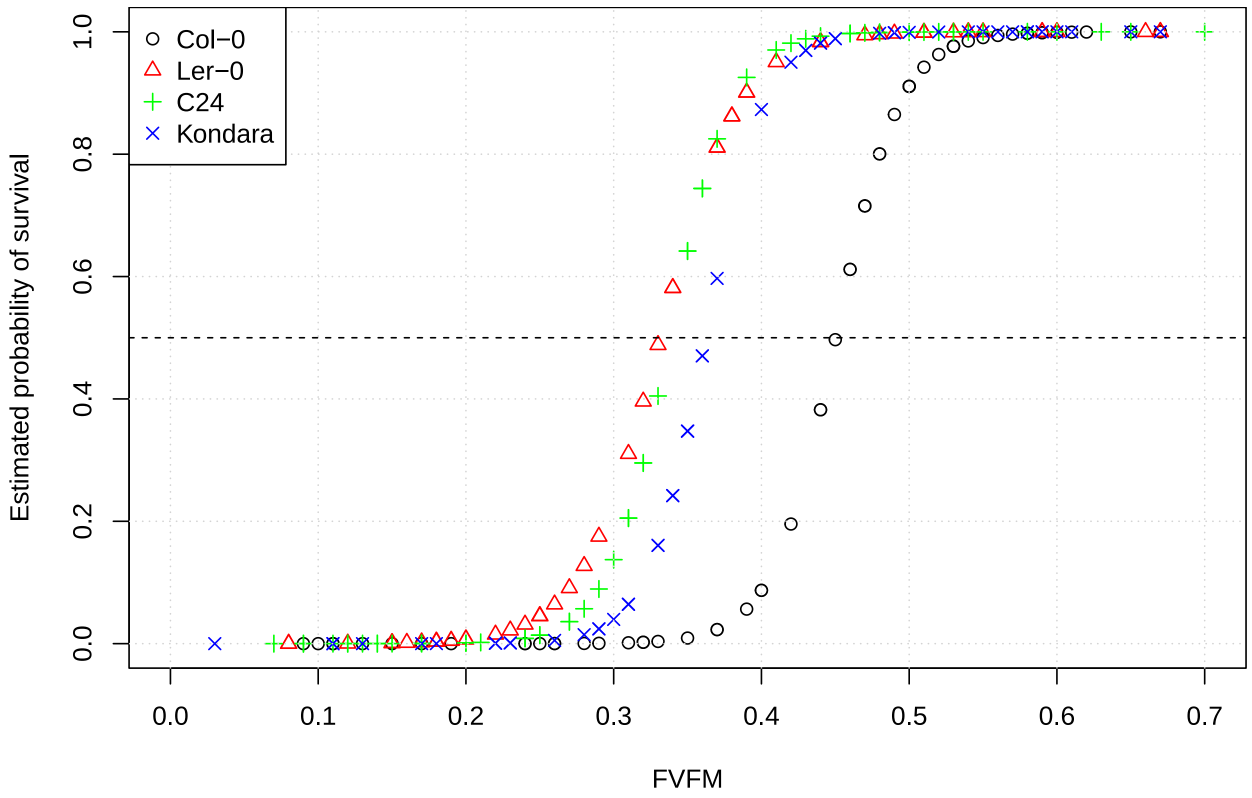

Figure 4. **Estimated probability of plant survival as function of the FVFM parameter in the four ecotypes.**

In Figure 4 we can see that, even when the rank at which the estimated probabilities have a sudden change from almost zero to almost one differ among ecotypes, being smaller and more alike in the Ler-0 and C24 ecotypes, intermediate in the Kondera ecotype and larger for the Col-0 ecotype, in all 4 cases this change is sudden, corroborating the fact that the estimate probability of survival quickly changes in a narrow rank of FVFM values. For practical proposes this means that to use the FVFM parameter to predict the survival phenotype it is necessary to perform a pilot calibration study to adequate to particular situations (ecotype, stress, etc.).

Using the criteria of predicting the survival phenotype by the 0.5 threshold of the estimated probability of survival produces very satisfactory results for all the 4 ecotypes, has shown in Table 3.

Table 3. Frequency of occurrence of predicted groups per ecotype in the sets of 60 data points per ecotype.

Ecotype	True Negative	False Negative	False Positive	True TruePos	Percent of Correct
Col-0	27	2	3	28	91.67
Ler-0	28	2	2	28	93.33
C24	29	1	1	29	96.67
Kondara	29	4	1	26	91.67

In Table 3 we can see that, by predicting the survival of an individual plant when the probability of survival estimated from the model was > 0.5, gives a large percentage ($\geq 91\%$) of correct predictions in all 4 ecotypes, while the rate of false negatives and false positives are small, in all cases $\leq 6\%$ (4/60).

The main objective of the statistical analyses presented here was to estimate the capability of the methodology to predict the results of independent data points by using models with parameters estimated from a *training* set.

Recapitulating, for each ecotype, $B = 1000$ different models using $n = 50$ data points were estimated, and the results were used to predict the phenotype on the $n = 10$ data points ignored in the estimation process. In Figure 3 we presented the results of the validation procedure for the Col-0 ecotype, showing that the rate of correct predictions is $\approx 90\%$ and discussing in detail the implications. Figures 5, 6 and 7 summarize the results of the validation process in the Ler-0, C24 and Kondra ecotypes, respectively.

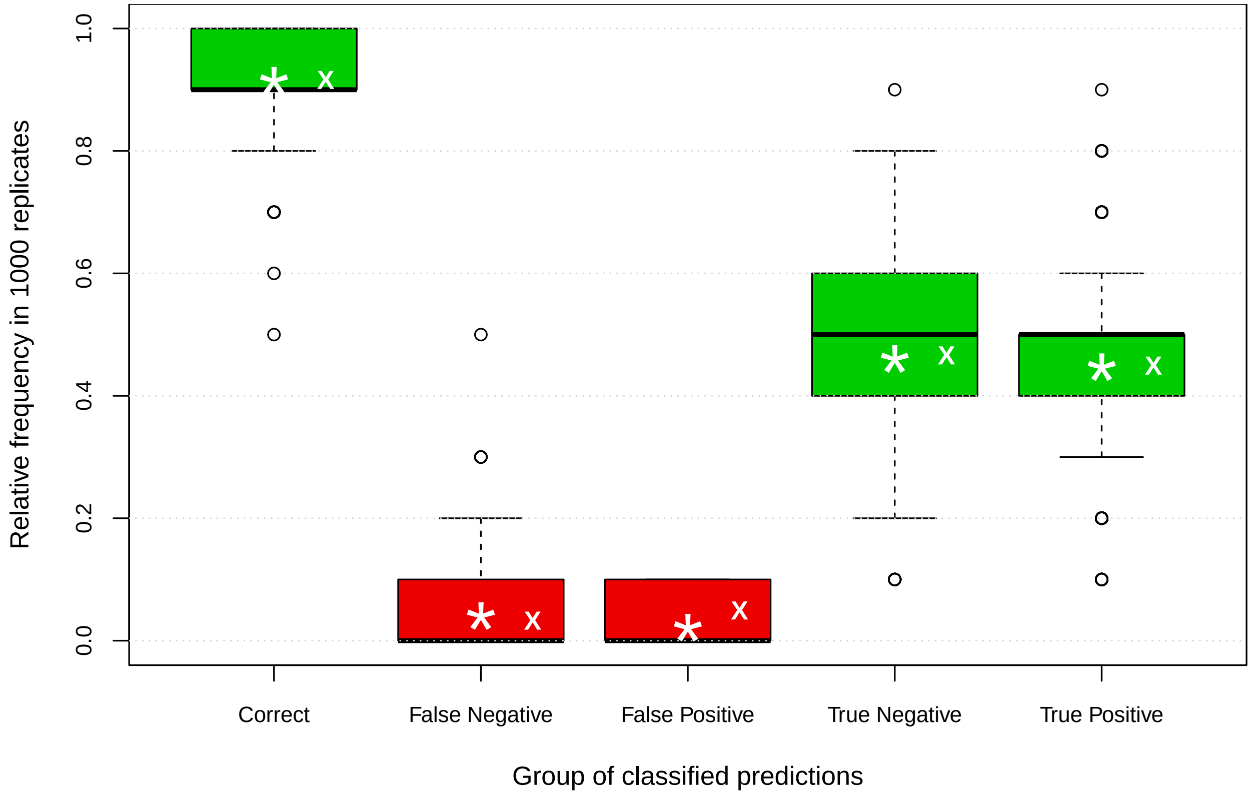

Figure 5. **Box plots for the validation of the procedure in the Ler-0 ecotype.** White asterisks show the mean of each frequency, while white "X" show the estimates in the model with all 60 data points.

From Figures 5, 6 and 7 we can see that the validation procedure produces correct predictions of independent observations in an average $\geq 90\%$ in all 4 ecotypes; in fact for the C24 ecotype, the median of correct predictions reaches 100%, while the rates of false positives and false negatives are reasonably low in all 4 ecotypes. Also, in all 4 ecotypes, the average rate of correct predictions of the validation procedure are very close to the estimated rates of correct predictions when using the full model (see white asterisks and crosses in the "Correct" distributions in figures 3, 5, 6 and 7).

Conclusion

The estimated FVFM parameter is a robust predictor of the probability of survival to drought stressed *Arabidopsis* plants, and thus can be used as a non-invasive method to predict plant survival. It is important to stress the fact that in all cases a pilot calibration experiment must be performed to estimate the suitable FVFM value for predicting the surviving of stressed plants under the particular conditions.

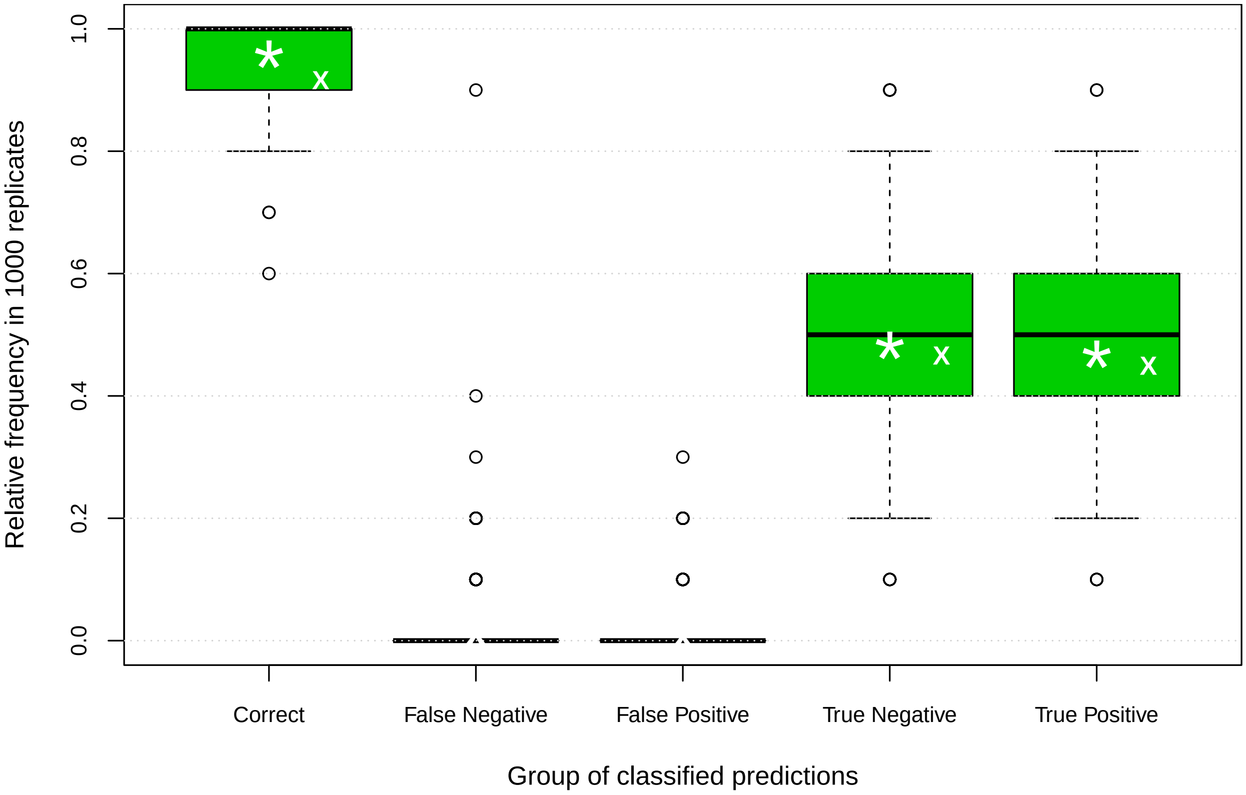

FIGURE 6. **Box plots for the validation of the procedure in the C24 ecotype.** White asterisks show the mean of each frequency, while white “X” show the estimates in the model with all 60 data points.

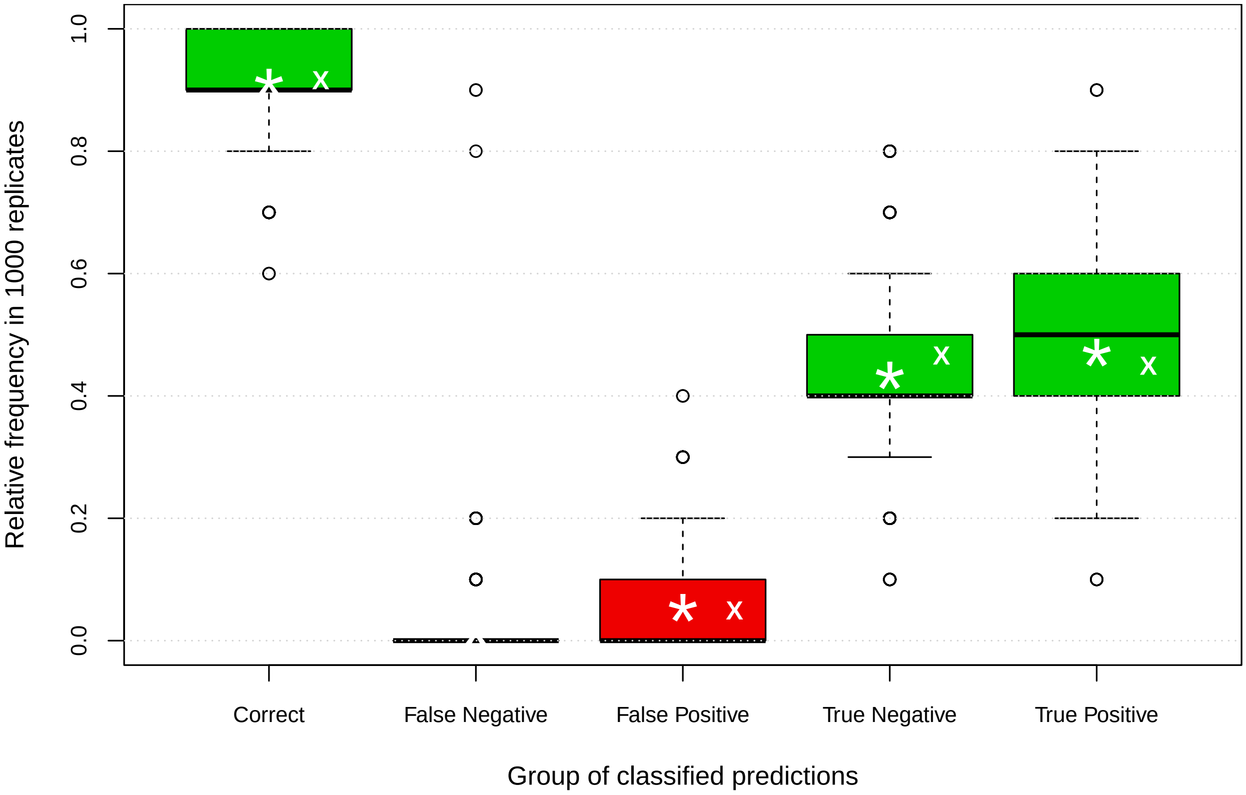

FIGURE 7. **Box plots for the validation of the procedure in the Kondara ecotype.** White asterisks show the mean of each frequency, while white "X" show the estimates in the model with all 60 data points .

References

Draper NR and Smith H (2014) *Applied regression analysis*, volume 326. John Wiley & Sons.

Harrell FE and Harrell FE (2001) *Chapter "Resampling, validating, describing, and simplifying the model"*. Springer. In: Regression Modeling Strategies: With Applications to Linear Models, Logistic Regression, and Survival Analysis.

R Core Team (2013) *R: A language and environment for statistical computing*. R Foundation for Statistical Computing, Vienna, Austria. URL `http://www.r-project.org`.
